# CpG ODN (K3)—toll-like receptor 9 agonist—induces Th1-type immune response and enhances cytotoxic activity in advanced lung cancer patients: a phase I study

**DOI:** 10.1186/s12885-022-09818-4

**Published:** 2022-07-07

**Authors:** Tomoyuki Otsuka, Sumiyuki Nishida, Takayuki Shibahara, Burcu Temizoz, Masanari Hamaguchi, Takayuki Shiroyama, Keiko Kimura, Kotaro Miyake, Haruhiko Hirata, Yumiko Mizuno, Mayu Yagita, Yusuke Manabe, Etsushi Kuroda, Yoshito Takeda, Hiroshi Kida, Ken J. Ishii, Atsushi Kumanogoh

**Affiliations:** 1 Department of Respiratory Medicine and Clinical Immunology, Osaka University Graduate School of Medicine, 2-2, Yamada-oka, Suita, Osaka, 565-0871 Japan; 2 Department of Medical Oncology, Osaka International Cancer Institute, 3-1-69, Otemae, Chuo-ku, Osaka, 541-8567 Japan; 3 Center for Vaccine and Adjuvant Research, National Institutes of Biomedical Innovation, Health and Nutrition, 7-6-8, Saito-Asagi, Ibaraki, Osaka, 567-0085 Japan; 4 Laboratory of Vaccine Science, Immunology Frontier Research Center, Osaka University, Suita, Osaka, Japan; 5 Division of Vaccine Science, Department of Microbiology and Immunology, The Institute of Medical Science, The University of Tokyo, 4-6-4, Shiraganedai, Minato-ku, Tokyo, 108-8639 Japan; 6 Department of Medical Innovation, Osaka University Hospital, 2-2, Yamada-oka, Suita, Osaka, 565-0871 Japan; 7 Department of Immunology, Hyogo College of Medicine, Nishinomiya, Hyogo Japan; 8 Department of Respiratory Medicine, National Hospital Organization Osaka Toneyama Medical Center, Toyonaka, Osaka, Japan; 9 Department of Immunopathology, Immunology Frontier Research Center, Osaka University, Suita, Osaka, Japan; 10 Integrated Frontier Research for Medical Science Division, Institute for Open and Transdisciplinary Research Initiatives, Osaka University, Suita, Osaka, Japan

**Keywords:** Cytosine-phosphate-guanine oligodeoxynucleotide, Toll-like receptor 9 agonist, Lung cancer, Immunotherapy, Dose-limiting toxicity

## Abstract

**Background:**

Cytosine-phosphate-guanine oligodeoxynucleotide (CpG ODN) (K3)—a novel synthetic single-stranded DNA immune adjuvant for cancer immunotherapy—induces a potential Th1-type immune response against cancer cells. We conducted a phase I study of CpG ODN (K3) in patients with lung cancer to assess its safety and patients’ immune responses.

**Methods:**

The primary endpoint was the proportion of dose-limiting toxicities (DLTs) at each dose level. Secondary endpoints included safety profile, an immune response, including dynamic changes in immune cell and cytokine production, and progression-free survival (PFS). In a 3 + 3 dose-escalation design, the dosage levels for CpG ODN (K3) were 5 or 10 mg/body via subcutaneous injection and 0.2 mg/kg via intravenous administration on days 1, 8, 15, and 29.

**Results:**

Nine patients (eight non-small-cell lung cancer; one small-cell lung cancer) were enrolled. We found no DLTs at any dose level and observed no serious treatment-related adverse events. The median observation period after registration was 55 days (range: 46–181 days). Serum IFN-α2 levels, but not inflammatory cytokines, increased in six patients after the third administration of CpG ODN (K3) (mean value: from 2.67 pg/mL to 3.61 pg/mL after 24 hours). Serum IFN-γ (mean value, from 9.07 pg/mL to 12.7 pg/m after 24 hours) and CXCL10 levels (mean value, from 351 pg/mL to 676 pg/mL after 24 hours) also increased in eight patients after the third administration. During the treatment course, the percentage of T-bet-expressing CD8^+^ T cells gradually increased (mean, 49.8% at baseline and 59.1% at day 29, *p* = 0.0273). Interestingly, both T-bet-expressing effector memory (mean, 52.7% at baseline and 63.7% at day 29, *p* = 0.0195) and terminally differentiated effector memory (mean, 82.3% at baseline and 90.0% at day 29, *p* = 0.0039) CD8^+^ T cells significantly increased. The median PFS was 398 days.

**Conclusions:**

This is the first clinical study showing that CpG ODN (K3) activated innate immunity and elicited Th1-type adaptive immune response and cytotoxic activity in cancer patients. CpG ODN (K3) was well tolerated at the dose settings tested, although the maximum tolerated dose was not determined.

**Trial registration:**

UMIN-CTR number 000023276. Registered 1 September 2016, https://upload.umin.ac.jp/cgi-open-bin/ctr/ctr_view.cgi?recptno=R000026649

**Supplementary Information:**

The online version contains supplementary material available at 10.1186/s12885-022-09818-4.

## Background

Cancer immunotherapy involves the induction or enhancement of a pre-existing host anti-tumor immune response to eradicate cancer cells. Immune checkpoint inhibition has recently created a new paradigm to improve the survival of patients with cancer, including non-small-cell lung cancer (NSCLC) [1–3]. However, the long-term beneficial effects of immune checkpoint inhibitors (ICIs) may be limited to approximately 20% of patients with advanced NSCLC [4–7]. ICIs can activate pre-existing immune cells, but they do not induce or mobilize them into the tumor microenvironment. Hence, they must be combined with immune adjuvants to further improve the prognosis of cancer patients [8]. Immune adjuvants include pathogen-associated molecular patterns or damage-associated molecular patterns that can activate various pattern-recognition receptors (PRRs), including toll-like receptors (TLRs), which are expressed on innate immune cells such as dendritic cells [9]. The activation of PRRs by immune adjuvants leads to the maturation of dendritic cells, which allows them to process antigens, present them to T cells, and produce type I IFNs and cytokines such as TNF-α and IL-12, resulting in the induction of antigen-specific immune responses [10].

Cytosine-phosphate-guanine oligodeoxynucleotide (CpG ODN) is a synthetic single-stranded DNA containing unmethylated CpG motifs commonly found in bacterial and viral genomes [11]. CpG ODNs are ligands for TLR9 that can activate both the TLR9-MyD88-IRF7 and TLR9-MyD88-NFκB signaling pathways to induce type I IFN and pro-inflammatory cytokine production by innate immune cells, leading to a Th1-type adaptive immune response [11]. Since Th1-type immune cells play a crucial role in anti-tumor immunity, CpG ODNs may contribute to the development of effective cancer immunotherapy.

CpG ODN (K3), a novel immune adjuvant developed by Ishii et al., has considerably high ability to stimulate human immune cells to proliferate and produce cytokines in vitro [12]. Recently, we conducted an investigator-initiated clinical trial of a CpG ODN (K3)-adjuvanted malaria vaccine in healthy volunteers. This vaccine was safe and tolerable and induced a more robust and substantially earlier immune response against the malaria antigen than the adjuvant-free vaccine did [13].

Herein, we present the results of our open-label, dose-escalation phase I trial to assess the safety profile and clinical and immune response of CpG ODN (K3) in patients with advanced lung cancer.

## Methods

### Study design

This study was an exploratory, prospective, dose-escalation phase I study on the safety and immunological effects of CpG ODN (K3) in patients with advanced lung cancer who had received standard platinum-based regimens. The study was approved by the institutional review board of Osaka University Hospital (reference number, 15507). Written informed consent was obtained from all patients before participation in the trial. The study was performed at Osaka University Hospital (Osaka, Japan) and registered with the University Hospital Medical Information Network (UMIN) Clinical Trials Registry (UMIN-000023276).

The primary endpoint was dose-limiting toxicities (DLTs) at each dose level. Secondary endpoints included progression-free survival (PFS), defined as the time from registration to documented disease progression or death from any cause; incidence of adverse events, assessed according to the Common Terminology Criteria for Adverse Events version 4.0; and immunological findings. Tumor response was based on investigator assessment according to the Response Evaluation Criteria in Solid Tumors version 1.1 [14].

Eligibility criteria were age between 20 and 79 years and histologically or cytologically confirmed NSCLC or small-cell lung cancer (SCLC). Eligible patients had undergone platinum-based chemotherapy alone or in combination with definitive radiation therapy. Additional inclusion criteria were no disease progression after treatments, an Eastern Cooperative Oncology Group performance status of 0–1, completion of prior treatment within 4 months, and adequate organ function.

### Treatment

**Fig. 1:**
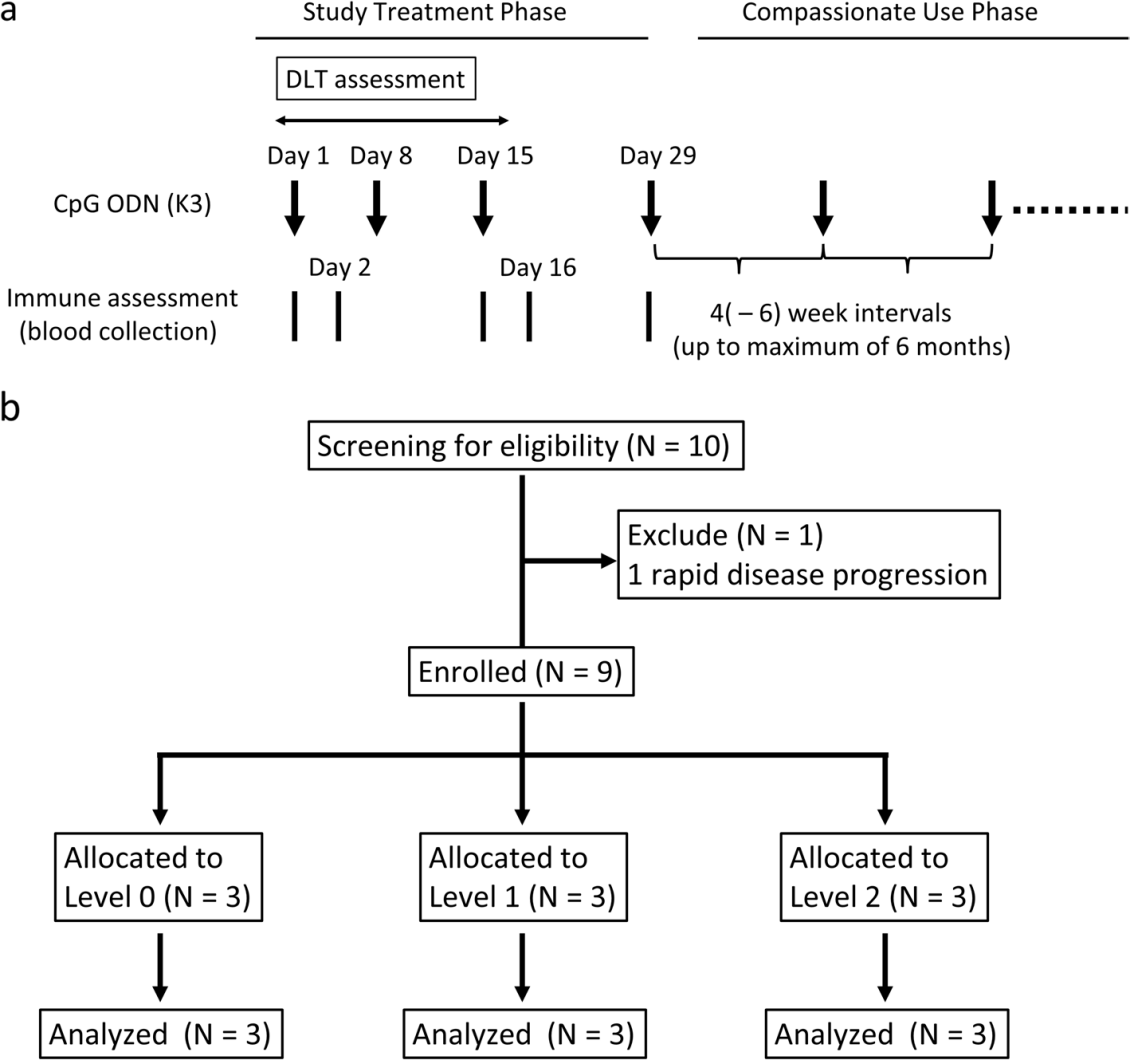
Schemas of treatment schedule (**a**) and CONSORT subject flow diagram (**b**)

Good manufacturing practice–grade CpG ODN (K3) (ATCG ACTC TCGA GCGT TCTC) synthesized by Gene Design (Ibaraki, Japan) was injected via subcutaneous (sc) or intravenous (iv) administration on days 1, 8, 15, and 29 (study treatment phase) and then every 4–6 weeks until a maximum of 6 months (compassionate use phase; Fig. 1a). Four basic doses of CpG ODN (K3) were scheduled: level 0, 5.0 mg/body sc; level 1, 10 mg/body sc; level 2, 0.2 mg/kg iv; and level 3, 0.6 mg/kg iv. Intrapatient dose escalation was not allowed during the DLT observation period.

Therapy was discontinued upon disease progression; severe adverse events, including grade 4 toxicities related to CpG ODN (K3); or request for withdrawal of consent. Concomitant treatment with bevacizumab maintenance therapy was allowed.

### Follow-up

Patients were followed up for their general condition and vital signs daily for the first 15 days on an inpatient basis. Thereafter, administration and assessment were performed on an outpatient basis. Blood tests were performed at baseline and after every administration (blood cell counts were also obtained on days 2 and 16 after a protocol amendment; Fig. 1a). Urine tests were performed at baseline and on days 15 and 29. Computed tomography was performed every 8 weeks until disease progression.

### Definition of DLTs

DLT was defined as the occurrence of any of the following, except for toxicities not related to CpG ODN (K3), from the first administration (day 1) to immediately after the third administration (day 15; Fig. 1a): (i) Grade 3 or 4 fever for 7 days or more; (ii) Grade 3 flu-like symptoms for 7 days or more; (iii) Grade 3 or 4 local skin reaction at administration sites; (iv) Grade 3 or 4 allergic reaction; (v) anaphylaxis; (vi) Grade 3 or 4 pneumonitis; (vii) Grade 3 or 4 neutropenia; (viii) Grade 3 or 4 thrombocytopenia; (ix) Grade 3 or 4 activated partial thromboplastin time prolongation; and (x) Grade 3 or greater non-hematological toxicity for 3 days or more, except for nausea or vomiting, anorexia, chills, hypotension, malaise or fatigue, and electrode abnormalities that were reversible with appropriate treatment.

### Estimation of MTD

For dose level escalation, a modified Fibonacci dose-escalation method (3 + 3 design) was used. At least three patients were treated at each dose level. If no DLT was observed, the dose was escalated to the next level. If one instance of DLT was observed among the three initial patients, an additional three patients were treated at the same dose level, and dose escalation was performed when no further DLTs were observed. If two of these six patients developed DLT, the dose level was estimated as the MTD.

### Immunological assays of peripheral blood cells and serum

For immune assessment, PBMCs and serum were collected before and 24 h after the first and third administration and before the fourth administration of CpG ODN (K3) during the study (Fig. 1a). PBMCs isolated from the blood samples were stored in liquid nitrogen until analysis. For the phenotyping of T cells by transcription factors, frozen PBMCs were defrosted with DNase I treatment and stained for the following surface markers: anti-human CD3-AF700 (clone: SK7), CD4-BV650 (clone: OKT4), CD8-BV510 (clone: SK1), CD45RA-FITC (clone: HI100), and CCR7-PE/Cy7 (clone: G043H7) antibodies and anti-human CD14-APC-Cy7 (clone: M5E2), CD19-APC-Cy7 (clone: HIB19), and CD56-APC-Cy7 (clone: HCD56) antibodies for dump gating (exclusion from the analysis) of monocytes, B cells, and NK cells, respectively, for 30 min. The cells were then permeabilized and fixed with the fixation buffer from the Foxp3/Transcription Factor Staining kit (eBioscience, USA) for 1 h. Next, they were washed with perm/wash buffer and stained for the intracellular proteins using anti-human Foxp3-APC (clone: PCH101) and T-bet-PE-Dazzle 594 (clone: 4B10) antibodies for 30 min. Data were acquired using a modified BD LSR II Fortessa (BD Biosciences, USA) and analyzed using FlowJo software. Human 48-plex Bioplex assay (Bio-Rad, USA) was performed to measure chemokine and cytokine levels in serum samples.

### Statistical analysis

PFS after registration was estimated using the Kaplan–Meier method to assess the time to disease progression or death. The cut-off date for data collection was March 24, 2020. Non-parametric Wilcoxon signed-rank test was used to calculate *p* values for the changes in immune cells and the levels of chemokines and cytokines. All analyses were performed using JMP Pro software, version 15 (SAS Institute Inc., Cary, NC, USA). Differences were considered statistically significant at *p* < 0.05.

## Results

### Patient characteristics

Between February 2017 and January 2019, we screened ten patients at Osaka University Hospital and enrolled nine in this study (Fig. 1b). Because of the enforcement of a new law on clinical research in Japan, we could complete only three of the four planned dosing levels (levels 0–2). The study was terminated on March 31, 2019. Patient characteristics at baseline are summarized in Table 1. Of the nine patients, eight had NSCLC (four adenocarcinomas, three squamous carcinomas, and one mucoepidermoid carcinoma), and one had SCLC. One patient (#08) discontinued first-line pembrolizumab therapy and then received platinum-based chemotherapy as a second-line treatment. The median time from the last treatment to enrollment in this study was 62 days. The median observation period after registration was 55 days (range: 46–181 days).

**Table 1:** Patient characteristics at baseline and individual clinical effects of CpG ODN (K3)

Dose level	Patient	Primary cancer	Age	Gender	PS	Smoking history	Stage	Prior therapy	Response to prior therapy	Time from the completion of prior therapy, (days)	No. of doses of CpG ODN (K3), n (n)^a^	Response to CpG ODN (K3)	PFS, (days)
5 mg sc	#01	NSCLC	62	F	0	Yes	IIIA	CRT	PR	45	3 (0)	non-CR/non-PD	398
	#02	NSCLC	59	M	0	No	IV	Chemotherapy	SD	33	4 (0)	PD	50
	#03	NSCLC	70	M	1	Yes	Recurrent	CRT	CR	109	9 (5)	non-CR/non-PD	891^b^
10 mg sc	#04	NSCLC	72	M	1	Yes	Recurrent	Chemotherapy	PR	88	8 (4)	non-CR/non-PD	330^c^
	#05	NSCLC	67	F	0	No	IV	Chemotherapy	PR	71	4 (0)	PD	49
	#06	SCLC	67	M	0	Yes	IV	Chemotherapy	PR	43	5 (1)	PD	72
0.2 mg/kg iv	#07	NSCLC	68	M	1	Yes	IV	Chemotherapy	SD	97	9 (5)	SD	294
	#08	NSCLC	72	M	1	Yes	IIIB	Chemotherapy	PR	36	4 (0)	SD	410^b^
	#09	NSCLC	77	M	1	Yes	IIIB	Chemotherapy	PR	62	4 (0)	SD	402^b^

NOTE: ^a^: The letter “n” refers to the total number of administrations of CpG ODN (K3) during the study (study treatment phase plus the compassionate use phase). The maximum number is 9. The number in parentheses “(n)” refers to a total number of administrations of CpG ODN (K3) in the compassionate use phase. The maximum number is 5. ^b^: These patients remained in progression-free survival on the cutoff date. These survival data were censored at the last observation. ^c^: This patient received an alternative anticancer therapy before radiological confirmation of disease progression. Survival data for this patient were censored at the beginning of the other treatment. Abbreviations: *sc* Subcutaneous injection, *iv* Intravenous administration, *M* Male, *F* Female, *PS* Performance status, *NSCLC* Non-small cell lung cancer, *SCLC* Small cell lung cancer, *CRT* Chemoradiotherapy, *PR* Partial remission, *SD* Stable disease, *CR* Complete remission, *PD* Progressive disease, *PFS* Progression-free survival

### Treatment course

Three patients each were treated at each dose level of CpG ODN (K3) (Table 1). One patient (#02) was concomitantly administered bevacizumab with CpG ODN (K3). None of the patients received any prophylactic medication for adverse events of CpG ODN (K3).

All patients except patient #01 completed four CpG ODN (K3) doses during the study treatment phase (Table 1). Patient #01 discontinued the study treatment after three doses of CpG ODN (K3) because of non-symptomatic exacerbation of pneumonitis, which was probably related to prior chemoradiation therapy (CRT). Four patients continued to receive CpG ODN (K3) in the compassionate use phase, and three of them completed the planned six-month treatment (Table 1).

### DLT and MTD of CpG ODN (K3)

No DLTs occurred in any of the patients at any dose level of CpG ODN (K3). We could not determine the MTD of CpG ODN (K3) at the dose settings in this study and estimated it to be greater than 10 mg sc or 0.2 mg/kg iv.

### Toxicity: treatment-related adverse events

Treatment-related adverse events (TrAEs) at each dose level of CpG ODN (K3) are summarized in Table 2. In total, 27 systemic adverse events were reported (Supplementary Table S1), of which 16 (59.3%) were determined to be TrAEs. All systemic TrAEs were of grade 1 or 2, were reversible without any treatment, and improved after the next administration. No symptoms or signs such as fever of > 38.0 °C or lymphadenopathy, which were assumed to occur as immune reactions related to CpG ODN (K3), were observed. No physical signs or symptoms suggesting the induction of autoimmunity were observed. No apparent differences in the incidence of each TrAE were noted between the dose groups.

**Table 2:** Treatment-related Adverse Events^a^

	Dose Level of CpG ODN (K3)					
	5 mg sc (*n* = 3)		10 mg sc (*n =* 3)		0.2 mg/kg iv (*n =* 3)	
Adverse event	Any Grade	Grade ≥ 3	Any Grade	Grade ≥ 3	Any Grade	Grade ≥ 3
White blood cell decreased	0	0	1	0	0	0
Neutrophil count decreased	1	0	1	0	0	0
Lymphocytes count decreased	1	0	1	0	0	0
Platelet count decreased	0	0	1	0	1	0
Eosinophil count increased	1	0	0	0	0	0
ALT increased	0	0	1	0	0	0
ALP increased	0	0	0	0	1	0
Hypocalcemia	0	0	1	0	0	0
Hypoalbuminemia	0	0	1	0	0	0
Urinary protein	0	0	1	0	0	0
Hematuria	0	0	0	0	1	0
Pneumonitis	1	0	0	0	0	0
Maculopapular rash	0	0	0	0	1	0
***Skin reactions at the local injection site***						
Redness	2	0	3	0	NE	NE
Induration	1	0	2	0	NE	NE

NOTE: ^a^Treatment-related adverse events were graded according to Common Terminology Criteria for Adverse Events (CTCAE) version 4.0

*Abbreviations*: *sc* Subcutaneous injection, *iv* Intravenous administration, *AST* Aspartate aminotransferase, *ALT* Alanine aminotransferase, *NE* Not evaluated

All patients of the sc groups except one (#02) exhibited mild local skin reactions at the injection sites within 48 h of the administration of CpG ODN (K3) (Table 2). There were no accompanying symptoms such as itching, and no treatment was required. No apparent differences in the severity of local skin reactions were noted between the two sc groups.

### Clinical effects of CpG ODN (K3)

Six of the nine patients (66.7%) had stable disease or non-CR/non-PD for 6 months as the best overall response (Table 1). The median PFS was 398 days (range, 49–891; Table 1 and Supplementary Fig. S1). No apparent association was noted between the dose level of CpG ODN (K3) and clinical effects. Notably, three patients (#03, #08, and #09) remained stable for over 1 year after the last administration of CpG ODN (K3).

### Immune assessment 1: effects on peripheral blood

**Fig. 2:**
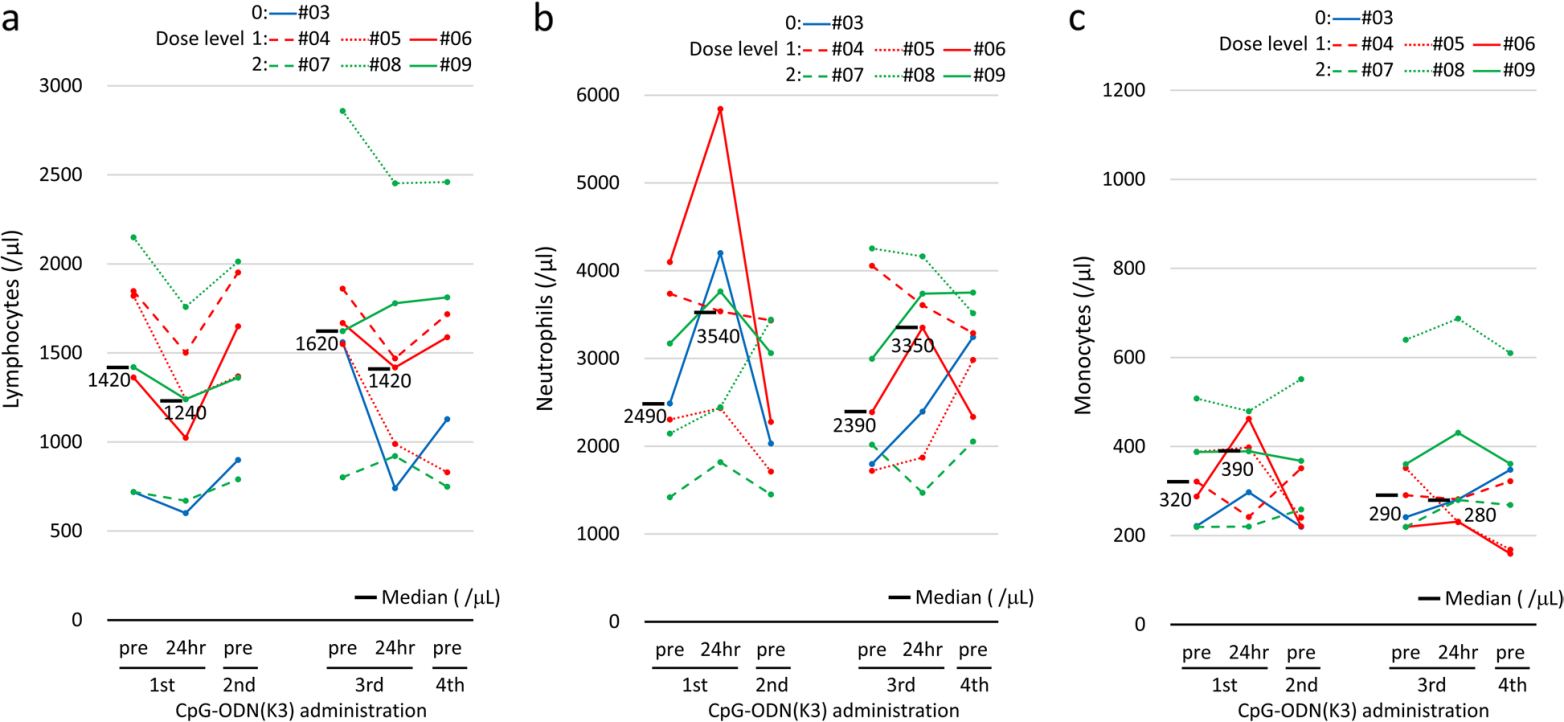
Dynamic changes in absolute lymphocyte count (**a**), absolute neutrophil count (**b**), and absolute monocyte count (**c**) in individual cases (#03 in the 5 mg sc cohort; #04, #05, and #06 in the 10 mg sc cohort; #07, #08, and #09 in the 0.2 mg/kg iv cohort). Peripheral blood samples were collected before (pre) the first, second, third, and fourth administration and 24 h after the first and third administration of CpG ODN (K3). This assessment was performed in seven patients because blood samples were not collected on days 2 and 16 in patients #01 and #02. Horizontal bars represent median values

We evaluated the effects of CpG ODN (K3) on peripheral blood cells such as lymphocytes, neutrophils, and monocytes. The lymphocyte counts decreased temporarily within 24 h of the administration of CpG ODN (K3) in all patients and returned to baseline by the next dosing schedule (Fig. 2a). In contrast, neutrophil counts appeared to increase temporarily within 24 h of the administration of CpG ODN (K3) (Fig. 2b), although no regular changes in monocyte counts were observed (Fig. 2c).

### Immune assessment 2: chemokine or cytokine production in response to CpG ODN (K3)

We evaluated the changes in serum cytokine and chemokine levels. Their levels increased to varying degrees within 24 h of CpG administration (Supplementary Fig. S2). We mainly focused on the time courses of IFN-α2, TNF-α, IFN-γ, and CXCL10 in response to CpG ODN (K3) (Table 3). The relative changes in these cytokines and chemokines during the acute phase within 24 h of treatment were more prominent after the third injection than after the first injection.

**Table 3:** Summary of cytokines and chemokines released or produced in response to CpG ODN (K3)

		Relative change before and 24 h after the administration of CpG ODN (K3) (%)^a^							
Dose Level	Pt.	1st administration				3rd administration			
		IFN-α2	TNF-α	IFN-γ	CXCL10	IFN-α2	TNF-α	IFN-γ	CXCL10
5 mg sc	#01	*▲24.8*	*▲37.6*	*▲26.3*	183.9	*▲31.4*	24.9	57.2	471.1
	#02	8.3	*▲1.92*	*▲13.9*	*▲3.3*	18.5	*▲15.9*	23.9	161.0
	#03	100.0	*▲12.2*	24.7	160.5	151.7	*▲20.9*	95.6	119.5
10 mg sc	#04	62.8	11.2	21.5	*▲8.7*	*▲38.6*	5.0	8.8	35.54
	#05	*▲59.4*	*▲22.7*	*▲36.3*	*▲35.5*	67.8	17.0	61.5	97.6
	#06	*▲26.6*	*▲10.8*	52.9	119.6	131.0	11.1	124.7	184.3
0.2 mg/kg iv	#07	*▲48.2*	*▲15.3*	*▲11.7*	*▲37.7*	100.0	*▲5.0*	24.9	2.59
	#08	485.1	32.3	*▲6.8*	72.5	*▲38.4*	*▲29.1*	*▲23.1*	*▲30.9*
	#09	*▲25.8*	*▲26.6*	*▲11.7*	*▲15.6*	191.7	10.0	19.4	53.2

^a^Comparison of IFN-α2, TNF-α, IFN-γ, or CXCL10 concentrations before and 24 h after the administration of CpG ODN (K3). Each value represents the relative change between before and 24 h after the administration of CpG ODN (K3), which was defined (post level – pre level)/pre level (%). *▲ italic* represents a negative value

*Abbreviations*: *sc* Subcutaneous injection, *iv* Intravenous administration

**Fig. 3:**
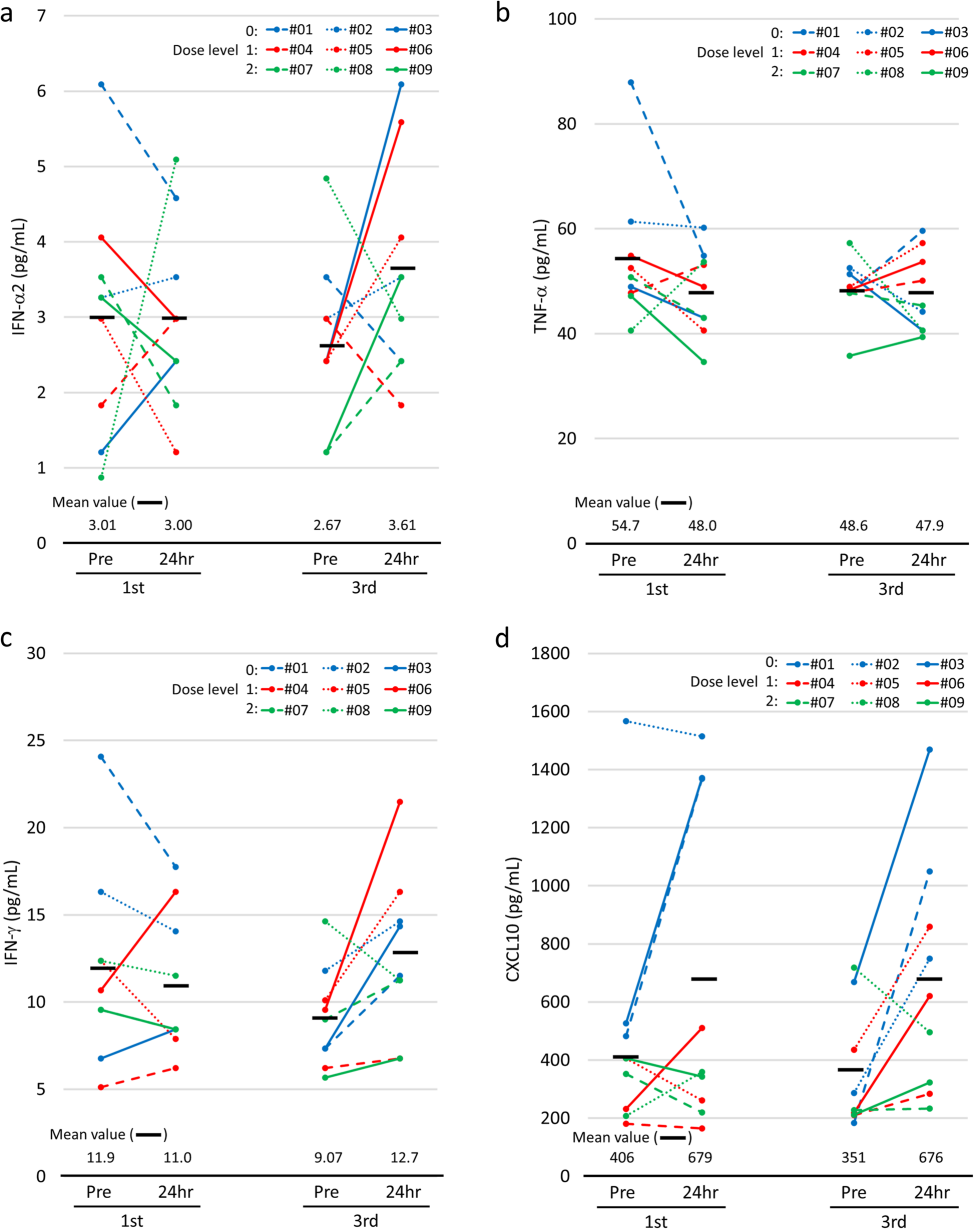
Dynamic changes in the levels of IFN-α2 (**a**), TNF-α (**b**), IFN-γ (**c**), and CXCL10 (**d**) in individual cases (#01, #02, and #03 in the 5 mg sc cohort; #04, #05, and #06 in the 10 mg sc cohort; and #07, #08, and #09 in the 0.2 mg/kg iv cohort). Peripheral blood samples were collected before (pre) the first and third administration and 24 h after the first and third administration of CpG ODN (K3). Horizontal bars represent median values

Type I IFNs and inflammatory cytokines are the markers of innate immune activation by CpG ODN (K3) [11]. In six patients, the serum levels of IFN-α2 increased mildly after the third administration of CpG ODN (K3) [mean IFN-α2 level, 2.67–3.61 pg/mL; median relative change, 67.8% (range: − 38.6 to 191.7%)], irrespective of the dose level or administration route (Fig. 3a and Table 3). In contrast, only a slight change in TNF-α levels was observed after the third CpG ODN (K3) dose in all patients [mean TNF-α level, 48.6–47.9 pg/mL; median relative change, 4.98% (range: − 29.1 to 24.9%)] (Fig. 3b and Table 3). Very few biologically meaningful changes were noted in the levels of other inflammatory cytokines, including IL-1β, IL-6, and IL-12 (Supplementary Fig. S2), suggesting that CpG ODN (K3) predominantly elicited type I IFN responses rather than inflammatory cytokine responses.

Next, we assessed the markers for adaptive immune activation, such as IFN-γ for Th1 response, IL-4 for Th2 response, and IL-17 for Th17 response. Eight patients had mild increases in serum IFN-γ level after the third dose [mean IFN-γ level, 9.07–12.7 pg/mL; median relative change, 24.9% (range: − 23.1 to 124.7%)] (Fig. 3c and Table 3). Five patients (#03, #05, #06, #07, and #09) had a mild-to-moderate increase in IFN-γ level in addition to a more than 50% relative increase in IFN-α2 (Table 3 and Fig. 3c). These results suggested that the increase in IFN-γ was related to the release of IFN-α2. However, the patients in the iv group had a weaker Th1 response than those in the sc groups. The mean relative changes in IFN-α2 in the iv and sc groups were 22.2% (range: 19.4–24.9%) and 76.4% (range: 23.9–124.7%), respectively. Unlike for IFN-γ, all patients showed minimal changes in serum IL-4 and IL-17 levels (Supplementary Fig. S2).

The serum levels of several chemokines, including CXCL10, CCL2, and CXCL9, increased temporarily within 24 h of CpG ODN (K3) administration (Fig. 3d and Supplementary Fig. S2). The serum level of CXCL10, also known as IFN-γ-induced protein 10, was increased in eight patients with relatively increased serum IFN-γ levels after the third dose [mean CXCL10 level, 351–676 pg/mL; median relative change, 97.6% (range: − 30.9 to 471.1%)] (Fig. 3d and Table 3). These responses were also considerably marked in the sc groups, but not dose-dependent.

### Immune assessment 3: induction of adaptive cellular immunity in response to CpG ODN (K3)

We evaluated the effect of CpG ODN (K3) on adaptive cellular immunity. Overall, no notable changes were observed in lymphocyte subsets of CD4- or CD8-positive CD3^+^ T cells and the percentages of their immune phenotypes (Supplementary Table S2).

**Fig. 4:**
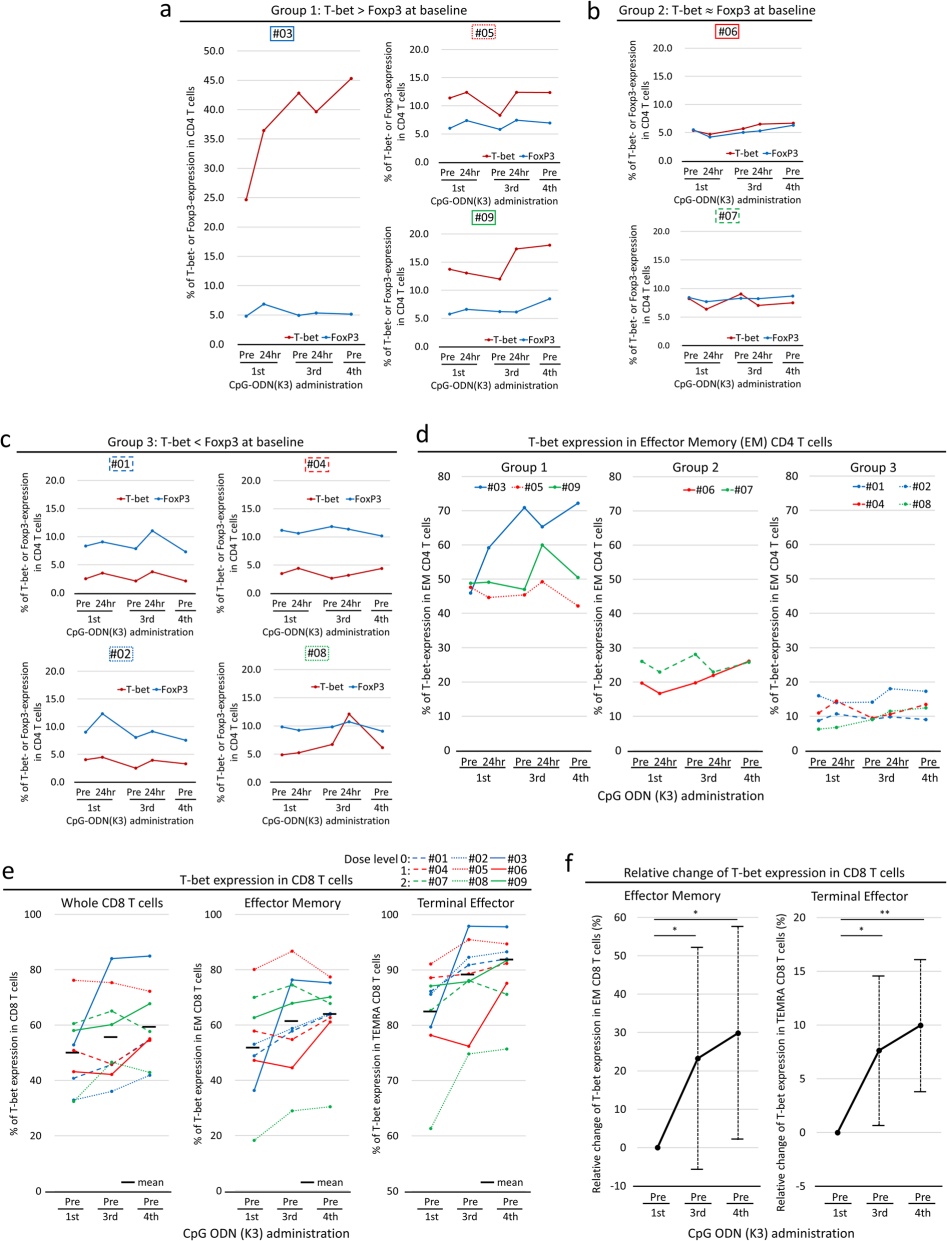
Dynamic changes in immune-related cells (**a**, **b**, **c**). Red and blue points indicate frequencies of T-bet- and Foxp3-positive CD4^+^ T cells, respectively, in individual cases. Peripheral blood samples were collected before (pre) the first, third, and fourth administration and 24 h after the first and third administration of CpG ODN (K3). Group 1: T-bet > Foxp3 at baseline (#03, #05, #09), Group 2: T-bet ≈ Foxp3 at baseline (#06, #07), Group 3: T-bet < Foxp3 at baseline (#01, #02, #04, #08). **d**, Each point indicates frequency of T-bet in EM CD4^+^ T cells in individual cases. **e**, Frequency of T-bet-positive CD8^+^ T cells in individual cases before (pre) the first, third, and fourth administration of CpG ODN (K3). (left) Whole CD8^+^ T cells, (middle) effector memory T cells, and (right) terminal effector cells. Horizontal bars represent median values. **f**, Relative changes in the T-bet expression in CD8^+^ T cells from baseline (Pre). (left) Effector memory T cells, and (right) terminal effector cells. Values are shown as the mean of nine patients. Error bars represent a 95% confidence interval. * *p* < 0.05, ** *p* < 0.01

First, we evaluated the functional characteristics of CD4^+^ T cells by analyzing T-bet and Foxp3 expression (Supplementary Fig. S3a). The percentages of T-bet^+^ and Foxp3^+^ CD4^+^ T cells were approximately 4–5% of the CD4^+^ T cells in PBMCs collected from two healthy subjects as controls (Supplementary Fig. S3b). Figure 4 (a–c) presents the classifications of patients into three groups based on T-bet or Foxp3 expression at baseline. In group 1, two patients (#03 and #09) exhibited a gradual increase in T-bet-expressing CD4^+^ T cells during the treatment course (24.7 and 13.7%, respectively, at baseline to 45.3 and 18.0% at day 29) (Fig. 4a). In contrast, in the other two groups, T-bet or Foxp3 expression in the CD4^+^ T cells changed minimally or fluctuated slightly during the treatment course (Fig. 4b-c). Immunophenotypically, especially in group 1, T-bet-expressing effector memory (EM) CD4^+^ T cells increased (Fig. 4d), whereas Foxp3-expressing EM CD4^+^ T cells remained almost unchanged during the treatment course (Supplementary Fig. S4).

Next, we evaluated the functional characteristics of CD8^+^ T cells by analyzing T-bet expression (Supplementary Fig. S3a). The percentage of T-bet^+^ CD8^+^ T cells was approximately 30% of the CD8^+^ T cells in the PBMCs collected from the two healthy subjects (Supplementary Fig. S3b). At baseline, all patients exhibited higher percentage of T-bet-expressing CD8^+^ T cells than those in the healthy subjects (Fig. 4e). During the treatment course, the percentage gradually increased (mean [range]: 49.8% [32.5–76.2] at baseline and 59.1% [41.9–84.9] at day 29, *p* = 0.0273; Fig. 4e). Immunophenotypically, both T-bet-expressing EM and terminally differentiated effector memory (TEMRA) CD8^+^ T cells significantly increased during the treatment course (mean [range]: EM, 52.7% [18.3–80.1] at baseline and 63.7% [30.5–77.5] at day 29, *p* = 0.0195; TEMRA, 82.3% [61.3–91.1] at baseline and 90.0% [75.7–97.8] at day 29, *p* = 0.0039; Fig. 4e). The relative changes in the T-bet-expressing EM and TEMRA CD8^+^ T cells significantly increased on days 15 and 29 (mean: EM, 23.3% at day 15, *p* = 0.0195, 29.8% at day 29, *p* = 0.0195; TEMRA, 7.6% at day 15, *p* = 0.0195, 10.0% at day 29, *p* = 0.0039; Fig. 4f).

### Case presentation (patient #03)

**Fig. 5:**
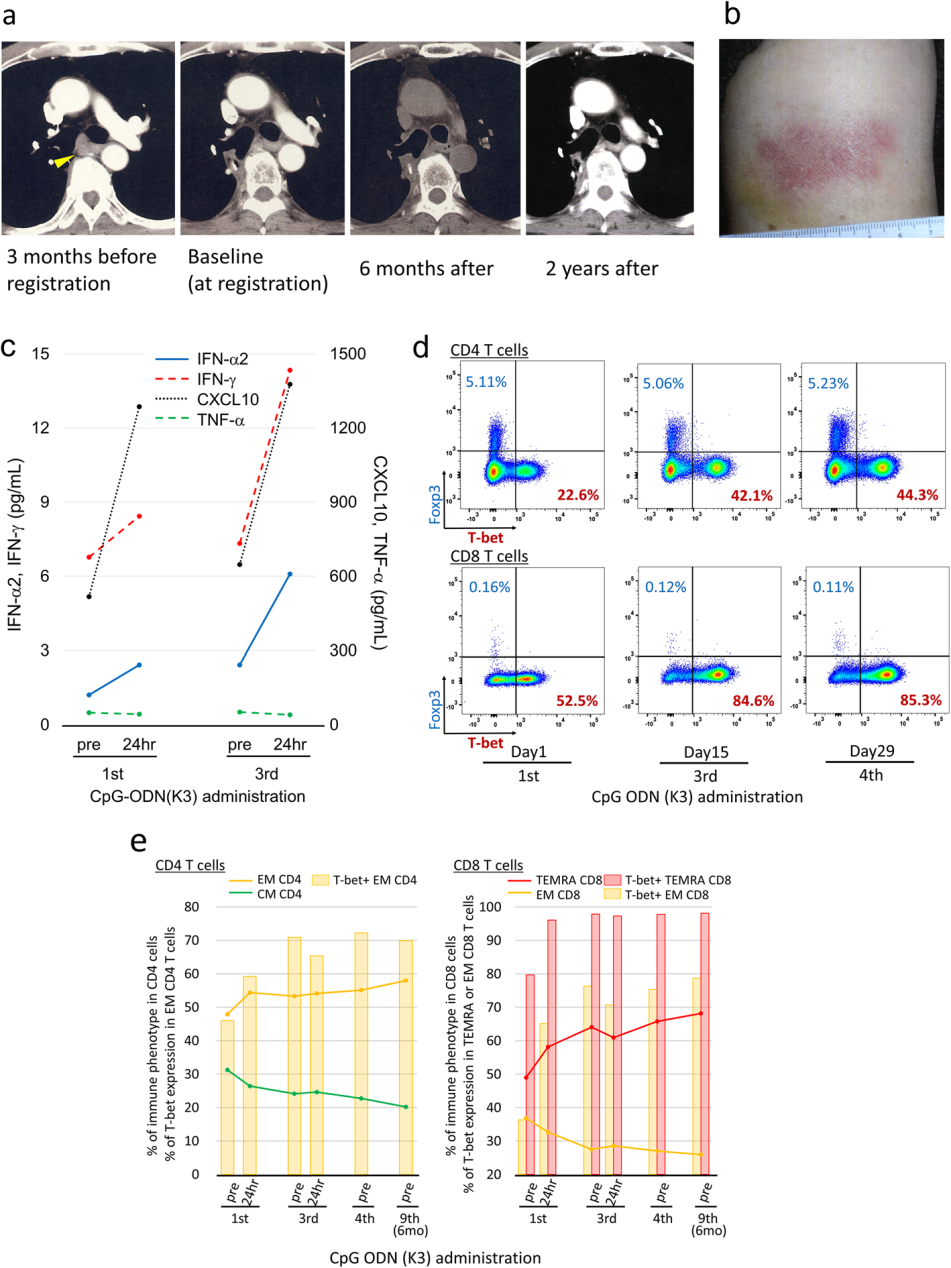
Clinical course and immunological monitoring in one case (#03). **a**, Chest computed tomography scan obtained three months before registration, at baseline, and six months and two years after the study treatment. The yellow triangle indicates lymph node metastasis. **b**, Redness of the skin about 5 cm in length at the injection site. **c**, Dynamic changes in the levels of IFN-α2, IFN-γ, TNF-α, and CXCL10 during the study treatment. Peripheral blood samples were collected before (pre) and 24 h after the first and third administration of CpG ODN (K3). **d**, Dot plots showing the expression of Foxp3 (y-axis) and T-bet (x-axis) in CD4^+^ and CD8^+^ T cells before the first, third, and fourth administration of CpG ODN (K3). **e**, (left) CD4^+^ T cells. The yellow bar represents the frequency of T-bet-positive EM CD4^+^ T cells. Yellow and green points indicate immunological phenotypes in EM and CM CD4^+^ T cells, respectively. (right) CD8^+^ T cells. Red and yellow bars represent the frequency of T-bet-positive cells in TEMRA and EM CD8^+^ T cells, respectively. Red and yellow points indicate immunological phenotypes in TEMRA and EM in CD8^+^ T cells, respectively

A 70-year-old man had relapsed NSCLC and received concurrent radiation therapy (Fig. 5a). He began receiving 5 mg CpG ODN (K3) via sc. He exhibited mild local skin reaction at the injection sites (Fig. 5b), but no serious systemic TrAEs. Notably, he showed disease stabilization for more than two years without any treatment (Fig. 5a).

Within 24 h of CpG ODN (K3) administration, his lymphocyte level temporally decreased (Fig. 2a) and serum IFN-α2, IFN-γ, and CXCL10 levels temporally increased (Fig. 5c). The T-bet expression in CD4^+^ T cells was approximately twice that of any other patients before the administration of CpG ODN (K3). During the treatment course, T-bet expression increased, whereas Foxp3 expression remained low (Fig. 5d). Notably, T-bet expression in EM CD4^+^ T cells increased after repeated CpG ODN (K3) administration and remained high for six months (Fig. 5e). T-bet expression also increased in CD8^+^ T cells (Fig. 5e). In CD8^+^ T cells, the percentage of TEMRA cells increased, whereas that of EM cells decreased, but both immune phenotypes showed a gradual increase in T-bet expression during the treatment course (Fig. 5e). The Th1-type cellular immune response sustained by CpG ODN (K3) possibly contributed to disease stabilization and led to better clinical results.

## Discussion

We conducted the first clinical study of CpG ODN (K3) in cancer patients. CpG ODN (K3) is expected to activate dendritic cells, leading to the initiation or enhancement of cancer immunity [15].

We observed no DLTs of CpG ODN (K3) at any dose level and could not determine the MTD of CpG ODN (K3) in our clinical setting. All systemic TrAEs were mild-to-moderate sporadic organ manifestations. No apparent differences in the profiles of systemic TrAEs were noted among the doses or routes of administration of CpG ODN (K3). No novel toxicities related to CpG ODNs, including autoimmune diseases, were found, and the toxicity profile of CpG ODN (K3) was similar to those of other CpG ODNs [16–25]. These results suggested that both sc and iv administration of CpG ODN (K3) were safe and well-tolerated. Several groups have shown that CpG ODNs cause TrAEs such as flu-like symptoms, lymphadenopathy, or coagulation abnormalities [16–25]. However, these adverse events were not noted in our patients. These results suggested that either the immunostimulatory and immunomodulatory functions of CpG ODN (K3) might differ from those of other CpG ODNs or the dose of CpG ODN (K3) used in this study could have failed to trigger these events.

One of the two patients treated with CRT as a prior treatment developed grade 1 interstitial pneumonitis after the third dose of CpG ODN (K3). This condition was probably a late effect of the prior radiation therapy. However, the immunity activated by CpG ODN (K3) might have exacerbated the condition. The other patient did not develop interstitial pneumonitis during the study and follow-up. Further clinical investigation is required.

As reported previously [16], we observed a transient decrease in lymphocytes. Although the detailed mechanism is unknown, this phenomenon could be related to the transient increase in chemokines after CpG ODN administration. In our study, serum CXCL-10 level increased transiently in response to CpG ODN (K3). CXCL-10 is a critical chemokine for effector T cell recruitment [16, 23, 25, 26]. The combination therapy of CpG ODN (K3) and ICIs might be a promising strategy for overcoming resistance to ICIs by facilitating the recruitment of effector T cells to the tumor site [26, 27].

Both sc and iv administration of CpG ODN (K3) led to the moderate production of IFN-α2 but no or slight increase in the serum levels of inflammatory cytokines, including TNF-α and IL-12. These results suggested that CpG ODN (K3) could predominantly activate the TLR9-MyD88-IRF7 signaling pathway in lung cancer patients. Type I IFNs produced by plasmacytoid dendritic cells enhance the cytotoxic activities of natural killer cells and CD8^+^ CTLs and promote the differentiation of CD4^+^ helper T cells, resulting in enhanced tumor-specific immune responses. The lack of serious TrAEs in this study could be because CpG ODN (K3) did not strongly induce inflammatory cytokines.

These immune responses were more remarkable after the third dose of CpG ODN (K3) than after the first dose. Studies using both mouse and primate models have shown that the adjuvant effects of CpG ODN (K3) were higher after multiple immunizations than after a single dose [28, 29]. Thus, we recommend a weekly schedule as the initial administration of CpG ODN (K3) for cancer immunotherapy to induce an early immune activation for targeting cancer cells.

In patients with greater T-bet expression than Foxp3 in CD4^+^ T cells (group 1), CD4^+^ T cells maintained or increased T-bet expression and immunophenotypically differentiated into EM-type T cells. Interestingly, in these patients, IFN-α2 increased notably in response to CpG ODN (K3) compared to that in other patients. These results suggested that CpG ODN (K3) could activate the Th1-type immune response in lung cancer patients. Thus, the frequency of CD4^+^ T cells with predominant T-bet expression may be a potential predictive biomarker for CpG ODN-based cancer immunotherapy.

T-bet expression represents the differentiation of cytotoxic CD8^+^ T cells because these cells require T-bet for IFN-γ and granzyme B production [30, 31]. The increase in IFN-γ could reflect the enhancement of cytotoxic activity by the differentiation into the effector phenotype. T cells are not directly responsive to CpG ODNs because of the lack of TLR9 expression. We presume that dendritic cells activated by CpG ODN (K3) stimulate CD8^+^ T cells through the interaction between the HLA/antigen peptide complex and T cell receptor, and then CD8^+^ T cells differentiate into effectors and exert a CTL response. These observations also suggest that CpG ODN (K3) induced a Th1-type immune response and enhanced cytotoxic activity in lung cancer patients.

This study designed two routes: sc and iv routes. The sc administration of CpG ODNs, which has been utilized by several clinical studies, activates localized dendritic cells at injection sites. Conversely, the iv administration of CpG ODNs is expected to systemically activate innate immunity, particularly immune cells in the tumor microenvironment, but the safety of this administration route has not been thoroughly evaluated. A preclinical study with a primate model showed that repeated administration of CpG ODN (K3) via the iv route was safe and increased serum levels of IFN-α2 [32]. In this study, we showed that 0.2 mg/kg by 30 min iv infusion of CpG ODN (K3) was as safe as the sc route and elicited Th1-type immune responses. However, these responses were comparable or slightly inferior to those via the sc route with regard to the production of systemic cytokines such as IFN-α2 and IFN-γ. This could be because of the rapid metabolic decomposition of CpG ODN in the blood [17]. Further investigation is necessary to determine the recommended dose and duration of iv administration of CpG ODN (K3).

This study has at least two limitations. First, we could not perform dose escalation in the iv cohort. The dose-dependent safety and immune effects of the iv administration and the MTD of CpG ODN (K3) remain unknown. Second, the number of patients was too small to allow the statistical assessment of the association between clinical outcome and immune response.

We recently reported the bioactivity of K3-SPG, a nanoparticulate CpG ODN (K3) wrapped by the nonagonistic Dectin-1 ligand schizophyllan [33]. This modification improves the drug delivery of CpG ODN (K3) to dendritic cells, thereby increasing type I IFN production. In a primate study, K3-SPG monotherapy induced a potent antigen-specific memory CTL response [32]. To conduct a clinical study of K3-SPG, we needed to confirm the clinical safety and potency of CpG ODN (K3) in cancer patients.

In the near future, we will plan to conduct the clinical trial of CpG ODN (K3) in combination with other cancer immunotherapies, including ICIs and cancer vaccines. These combinations are expected to additively enhance the anti-tumor immune response against lung cancer and other solid cancers. On the other hand, the combination immunotherapies may also emerge immune-related adverse events unexpected. We will also conduct the first clinical study to evaluate the safety and efficacy of K3-SPG in patients with solid cancers and then design a combination therapy with other cancer immunotherapies.

## Conclusions

CpG ODN (K3) is safe as an immune adjuvant in patients with advanced lung cancer. The administration of CpG ODN (K3) via either sc or iv route activated innate immunity, leading to the elicitation of Th1-type adaptive immune responses and enhancement of cytotoxic activity. Further clinical studies are needed to determine whether cancer patients have beneficial clinical outcomes with CpG ODN (K3) alone or in combination with other agents, especially ICIs.

## Supplementary Information

**Additional file 1: Supplementary Table S1.** Adverse Events. **Supplementary Table S2.** Immune phenotypes of CD4^+^ and CD8^+^ T cells.

**Additional file 2: Supplementary Fig. S1.** Kaplan–Meier curves for progression-free survival (PFS). **Supplementary Fig. S2.** Analysis of chemokines and cytokines. **Supplementary Fig. S3.** Gating strategy for immune phenotyping and functional characteristics of T cells. **Supplementary Fig. S4.** Foxp3 expression in effector memory of CD4^+^ T cells in individual cases.

## Data Availability

All data generated or analysed during this study are included in this published article and its supplementary information files.

## Abbreviations

CpG ODN: Cytosine-phosphate-guanine oligodeoxynucleotide
DLT: Dose-limiting toxicity
EM: Effector memory
ICI: Immune checkpoint inhibitor
iv: Intravenous
MTD: Maximum tolerated dose
NSCLC: Non-small-cell lung cancer
PFS: Progression-free survival
PRR: Pattern-recognition receptor
sc: Subcutaneous
SCLC: Small-cell lung cancer
TEMRA: Terminally differentiated effector memory
TLR: Toll-like receptor
TrAE: Treatment-related adverse event
UMIN: University Hospital Medical Information Network
