# Supplementary material for: CpG ODN (K3)—toll-like receptor 9 agonist—induces Th1-type immune response and enhances cytotoxic activity in advanced lung cancer patients: a phase I study
Source: BMC Cancer. 2022 Jul 7;22:744. doi: 10.1186/s12885-022-09818-4 (PMC9264631; doi:10.1186/s12885-022-09818-4)
Supplement: Supplementary file 1 — Additional file 1: Supplementary Table S1. Adverse Events. Supplementary Table S2. Immune phenotypes of CD4+ and CD8+ T cells. [file 12885_2022_9818_MOESM1_ESM.pdf]

Supplementary Table S1. Adverse Events.

Adverse event	Dose level of CpG ODN (K3)					
	5 mg sc (n = 3)		10 mg sc (n = 3)		0.2 mg/kg iv (n = 3)	
	Any Grade	Grade ≥3	Any Grade	Grade ≥3	Any Grade	Grade ≥3
White blood cell decreased	1	0	1	0	0	0
Neutrophil count decreased	1	0	1	0	0	0
Lymphocytes count decreased	2	0	1	0	0	0
Platelet count decreased	0	0	1	0	1	0
Eosinophil count increased	1	0	0	0	0	0
ALT increased	0	0	1	0	0	0
ALP increased	0	0	0	0	1	0
Hypocalcemia	0	0	1	0	0	0
Hypoalbuminemia	0	0	1	0	0	0
Urinary protein	0	0	1	0	0	0
Hematuria	0	0	3	0	1	0
Pneumonitis	2	0	0	0	0	0
Maculopapular rash	0	0	0	0	1	0
Constipation	1	0	0	0	0	0
Conjunctivitis	1	0	0	0	0	0
Dysgeusia	1	0	0	0	0	0
Bronchial infection	0	0	1	0	0	0
Tinnitus	0	0	1	0	0	0
Skin reactions at the local injection site						
Redness	2	0	3	0	NE	NE
Induration	1	0	2	0	NE	NE

NOTE: Adverse events were graded according to Common Terminology Criteria for Adverse Events (CTCAE) version 4.0.

Abbreviations: sc, subcutaneous injection; iv, intravenous administration; AST, aspartate aminotransferase; ALT, alanine aminotransferase; NE, not evaluated.

Supplementary Table S2. Immune phenotypes of CD4$^+$ and CD8$^+$ T cells

	Day 1 (Baseline)	Day 15		Day 29	
	Median [min, max]	Median [min, max]	*p*-value*	Median [min, max]	*p*-value*
No. of patients	n = 9	n = 9		n = 9	
CD4 T$^+$ cells					
Total CD4, %	64.3 [49.0, 80.9]	62.7 [48.0, 82.2]	0.9102	64.2 [49.8, 77.7]	0.6797
Immune phenotype					
Naïve, %	42.4 [17.7, 57.5]	43.6 [17.6, 65.4]	0.5156	39.8 [16.6, 55.4]	0.8203
Central Memory, %	29.5 [20.0, 49.9]	29.9 [17.5, 53.6]	0.7422	26.5 [22.5, 50.1]	1.0000
Effector Memory, %	23.7 [16.8, 47.9]	20.4 [15.1, 53.3]	0.2656	25.7 [16.9, 55.1]	0.7344
Terminal Effector, %	0.93 [0.31, 5.51]	0.79 [0.25, 6.56]	0.8438	0.85 [0.41, 5.60]	0.5547
CD8 T$^+$ cells					
Total CD8, %	32.9 [13.9, 42.3]	32.8 [12.8, 43.2]	0.9102	33.2 [17.4, 42.3]	0.7539
Immune phenotype					
Naïve, %	17.7 [5.61, 31.5]	16.4 [4.96, 35.2]	0.8203	19.3 [4.11, 26.4]	0.5156
Central Memory, %	8.57 [1.37, 21.3]	8.15 [1.55, 22.7]	0.4961	6.89 [3.10, 17.6]	0.1289
Effector Memory, %	39.4 [24.9, 58.2]	34.3 [22.3, 57.4]	0.1289	38.5 [23.0, 59.7]	0.0781
Terminal Effector, %	30.3 [14.1, 49.2]	35.3 [16.5, 64.1]	0.4453	41.2 [17.2, 65.8]	0.1367

NOTE: Total CD4 or CD8 T cells were defined as CD4- or CD8-positive CD3 T cells, respectively. Each immune phenotype was defined as follows: naïve, CD45RA+ CCR7+; central memory, CD45RA- CCR7+; effector memory, CD45RA- CCR7-; terminal effector, CD45RA+ CCR7-. *: Values at days 15 or 29 were compared to baseline values. The *p*-value was calculated using non-parametrical Wilcoxon signed-rank test.
