# Supplementary material for: CpG ODN (K3)—toll-like receptor 9 agonist—induces Th1-type immune response and enhances cytotoxic activity in advanced lung cancer patients: a phase I study
Source: BMC Cancer. 2022 Jul 7;22:744. doi: 10.1186/s12885-022-09818-4 (PMC9264631; doi:10.1186/s12885-022-09818-4)

Supplementary Figure S1.

Kaplan–Meier curves for progression-free survival (PFS).

PFS was defined as the time from enrollment to documented disease progression or death due to any cause. Median PFS was 398 days

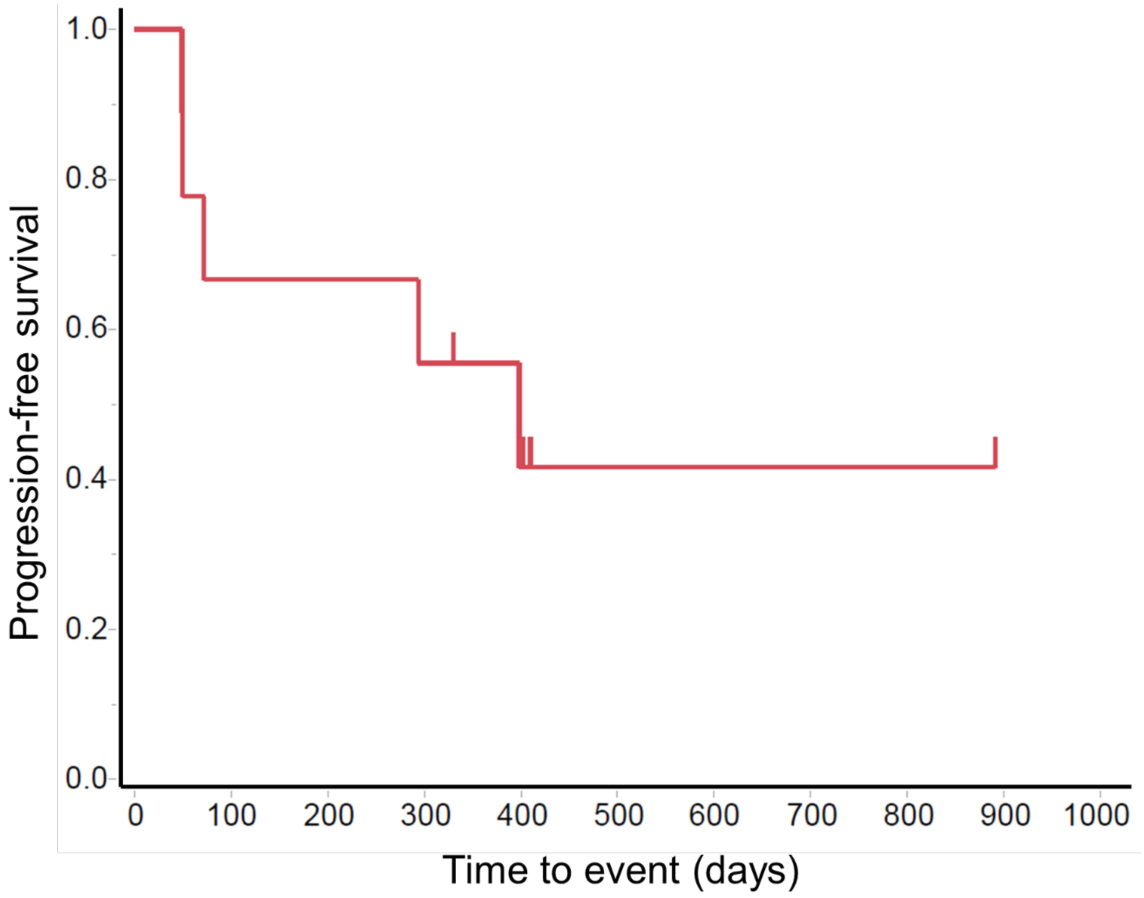

Supplementary Figure S2. Analysis of chemokines and cytokines.

The serum levels of several chemokines and cytokines were analyzed using BioPlex. Serum was collected before and 24 h after the first and third administration of CpG ODN (K3).

a. CXCL1. **b.** CXCL9. **c.** CCL2. **d.** CCL3. **e.** CCL4. **f.** CCL5. **g.** CCL11. **h.** CCL27. **i.** IL-1α. **j.** IL-1β. **k.** IL-1Ra. **l.** IL-2Rα. **m.** IL-4. **n.** IL-6. **o.** IL-8. **p.** IL-9. **q.** IL-10. **r.** IL-13. **s.** IL-16. **t.** IL-17. **u.** IL-18. **v.** TRAIL. **w.** FGF-basic. **x.** M-CSF. **y.** PDGF-bb. **z.** TNF-β. **aa.** HGF. **ab.** MIF. **ac.** β-NGF. **ad.** SCF. **ae.** SCGF-β. **af.** SDF-1α. **ag.** VEGF.

The data of CCL7, IL-2, IL-3, IL-5, IL-7, IL-12 (p70), IL-12 (p40), IL-15, G-CSF, GM-CSF, and LIF were not shown because the majority of results were less than the lower limit of quantification. Line graphs represents the change in chemokines and cytokines in each patient. Blue lines: dose level 0, Red line: dose level 1, and Green line: dose level 2

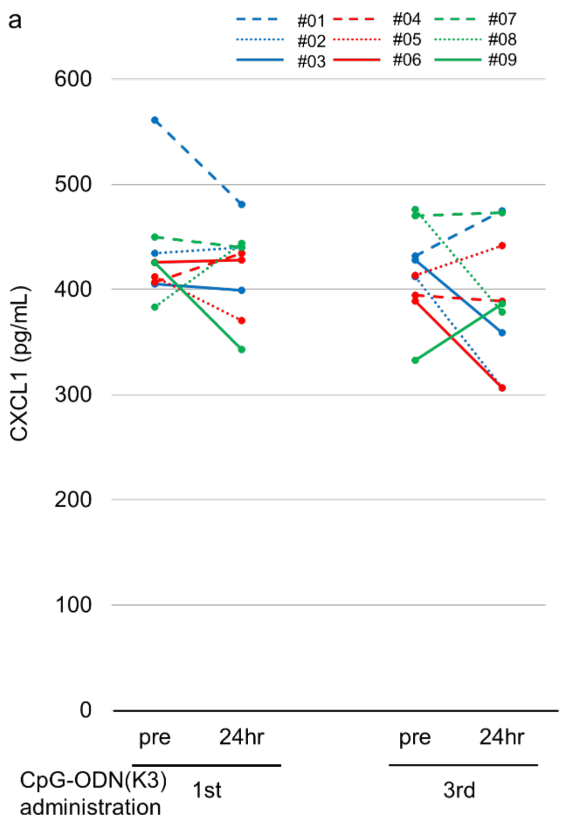

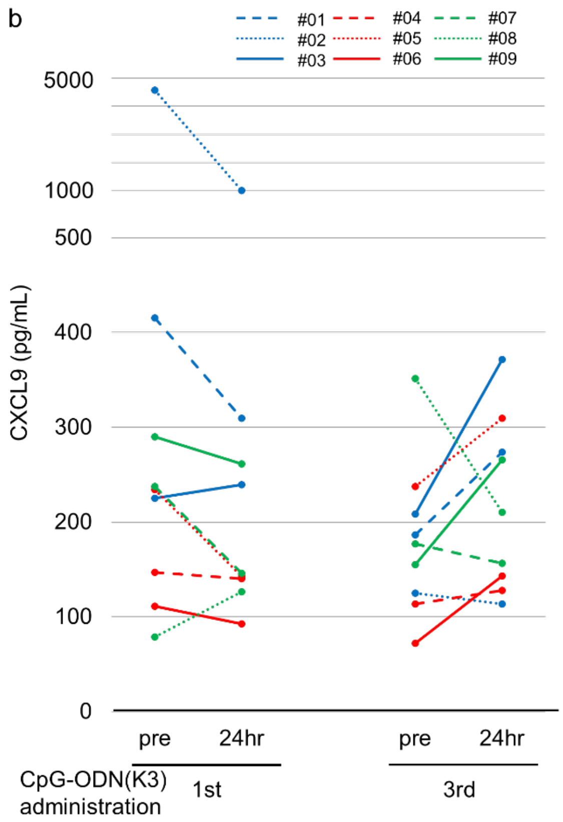
b
#01
#02
#03
#04
#05
#06
#07
#08
#09
5000
1000
500
400
300
200
100
0
CXCL9 (pg/mL)
pre
24hr
pre
24hr
CpG-ODN(K3) administration
1st
3rd

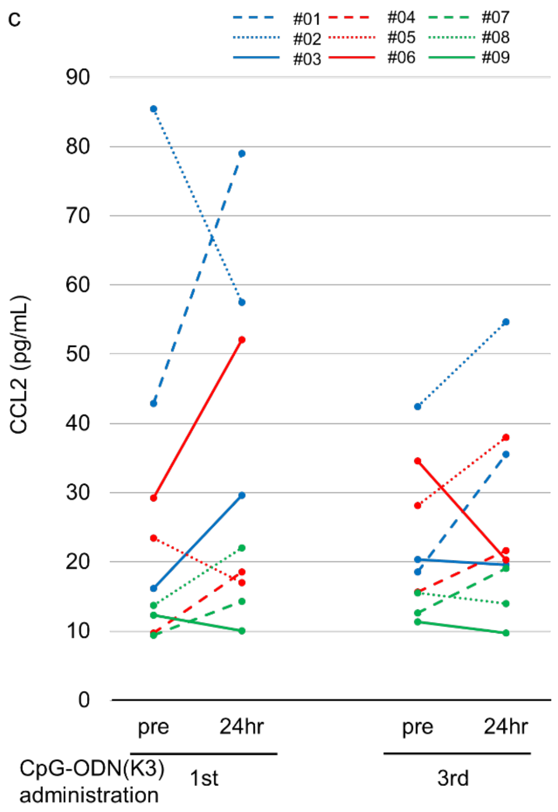
c
#01
#02
#03
#04
#05
#06
#07
#08
#09
90
80
70
60
50
40
30
20
10
0
CCL2 (pg/mL)
pre
24hr
pre
24hr
CpG-ODN(K3) administration
1st
3rd

d

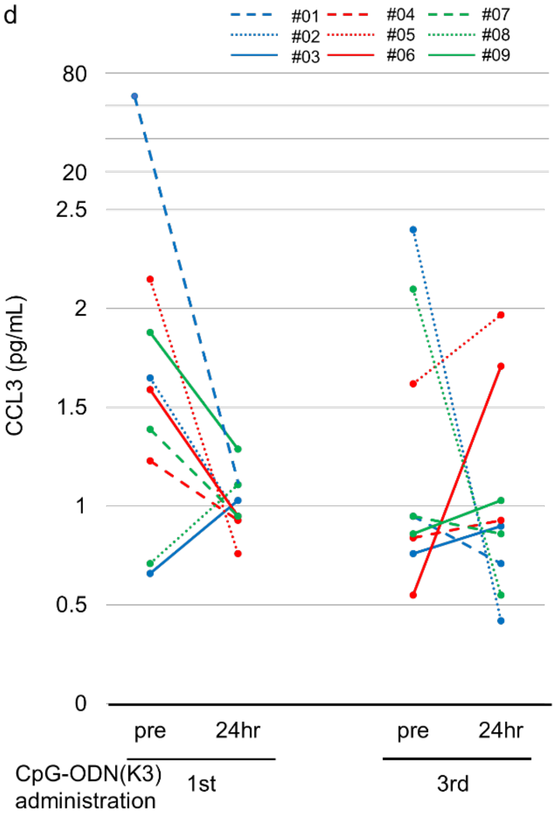
#01
#02
#03
#04
#05
#06
#07
#08
#09
80
20
2.5
2
1.5
1
0.5
0
CCL3 (pg/mL)
pre
24hr
pre
24hr
CpG-ODN(K3)
administration
1st
3rd

e

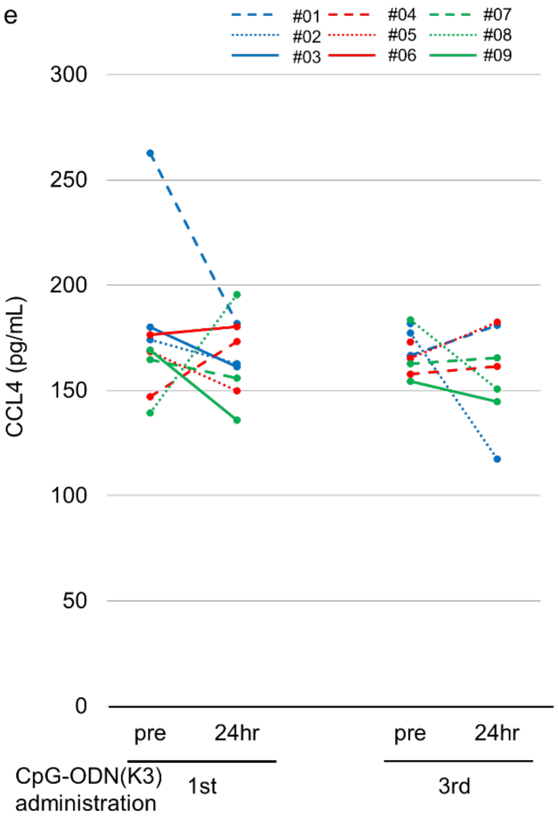
#01
#02
#03
#04
#05
#06
#07
#08
#09
300
250
200
150
100
50
0
CCL4 (pg/mL)
pre
24hr
pre
24hr
CpG-ODN(K3)
administration
1st
3rd

f

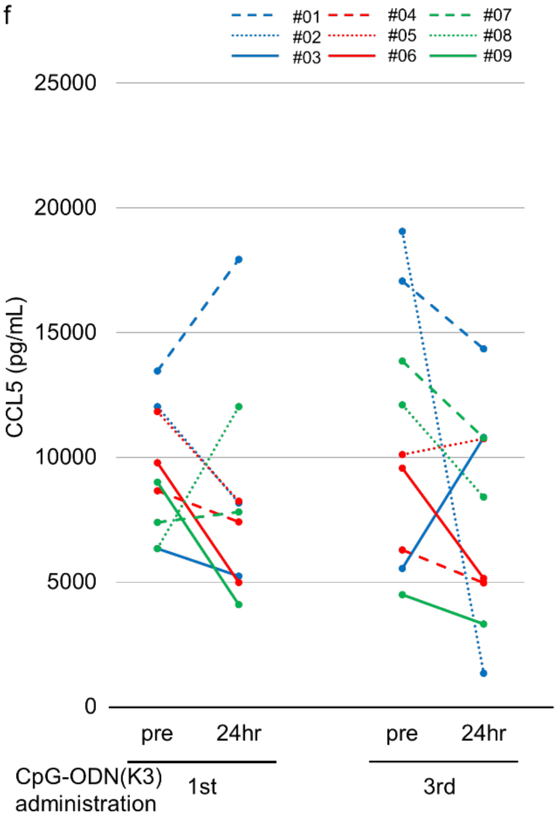

#01
#02
#03
#04
#05
#06
#07
#08
#09
CCL5 (pg/mL)
25000
20000
15000
10000
5000
0
pre
24hr
pre
24hr
CpG-ODN(K3) administration
1st
3rd

g

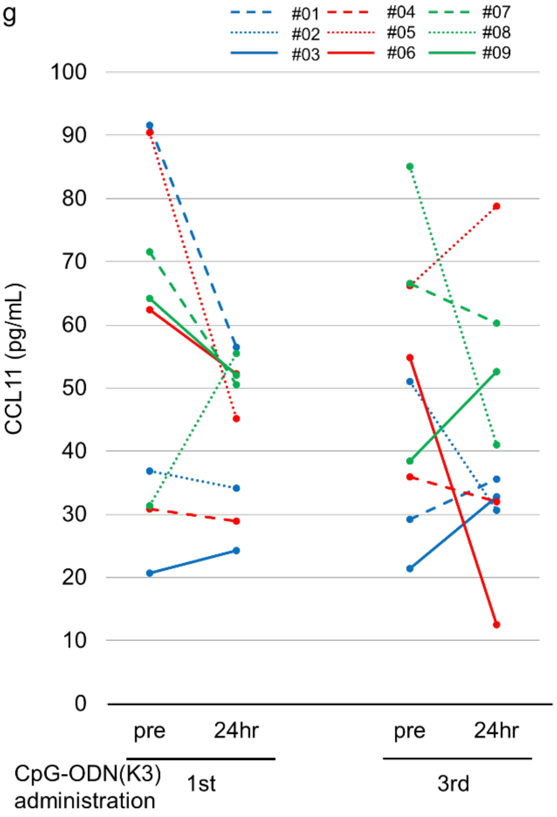

#01
#02
#03
#04
#05
#06
#07
#08
#09
CCL11 (pg/mL)
100
90
80
70
60
50
40
30
20
10
0
pre
24hr
pre
24hr
CpG-ODN(K3) administration
1st
3rd

h

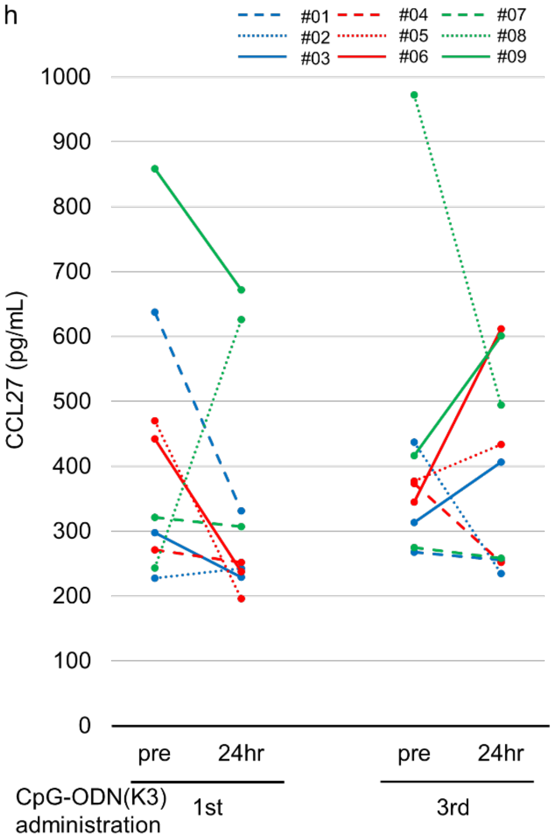
#01
#02
#03
#04
#05
#06
#07
#08
#09
1000
900
800
700
600
500
400
300
200
100
0
CCL27 (pg/mL)
pre
24hr
pre
24hr
CpG-ODN(K3) administration
1st
3rd

i

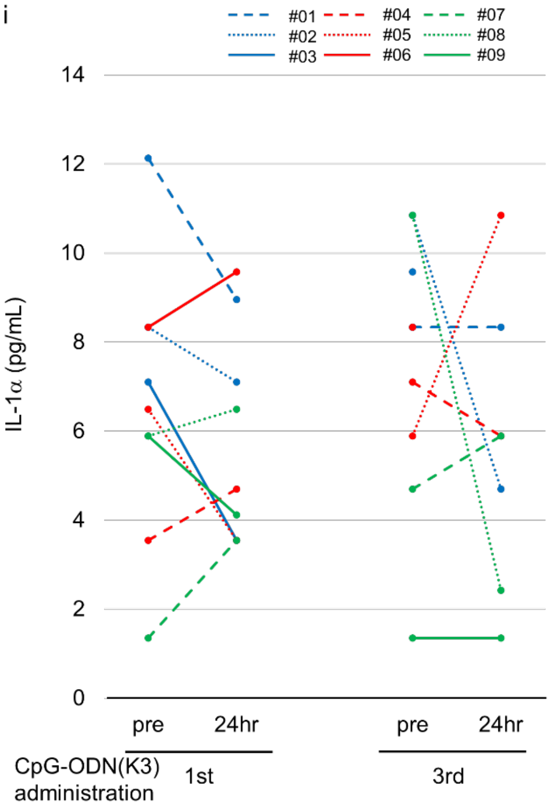
#01
#02
#03
#04
#05
#06
#07
#08
#09
14
12
10
8
6
4
2
0
IL-1α (pg/mL)
pre
24hr
pre
24hr
CpG-ODN(K3) administration
1st
3rd

j

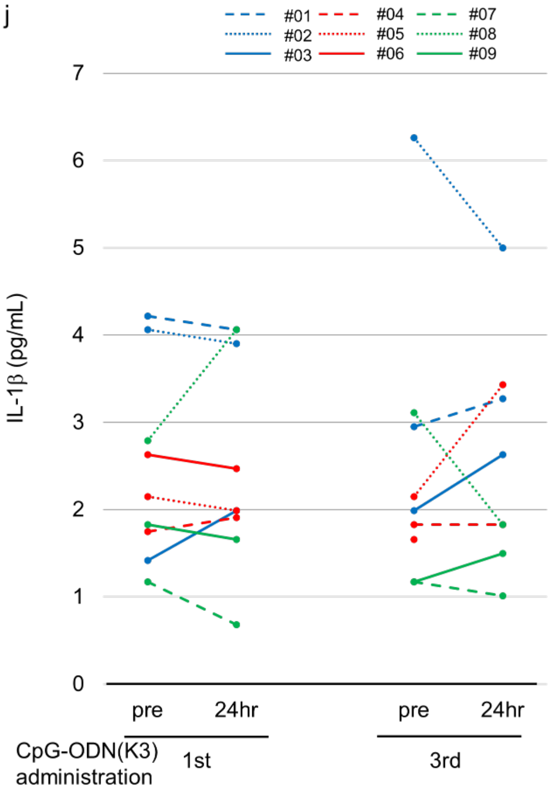

#01
#02
#03
#04
#05
#06
#07
#08
#09
IL-1β (pg/mL)
7
6
5
4
3
2
1
0
pre
24hr
pre
24hr
CpG-ODN(K3)
administration
1st
3rd

k

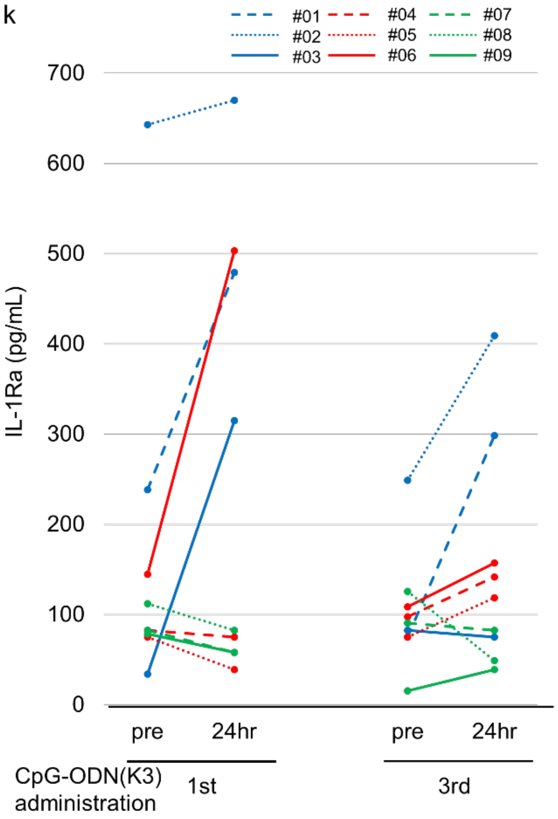

#01
#02
#03
#04
#05
#06
#07
#08
#09
IL-1Ra (pg/mL)
700
600
500
400
300
200
100
0
pre
24hr
pre
24hr
CpG-ODN(K3)
administration
1st
3rd

l

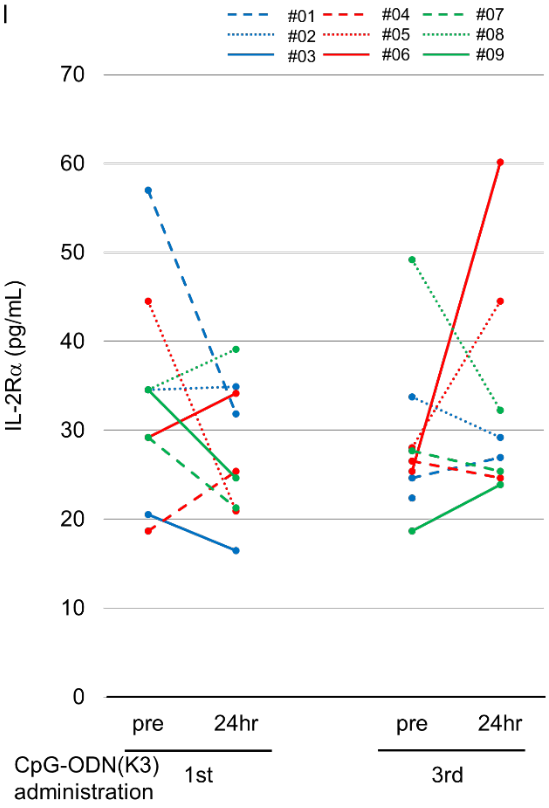
#01
#02
#03
#04
#05
#06
#07
#08
#09
70
60
50
40
30
20
10
0
IL-2Rα (pg/mL)
pre
24hr
pre
24hr
CpG-ODN(K3)
administration
1st
3rd

m

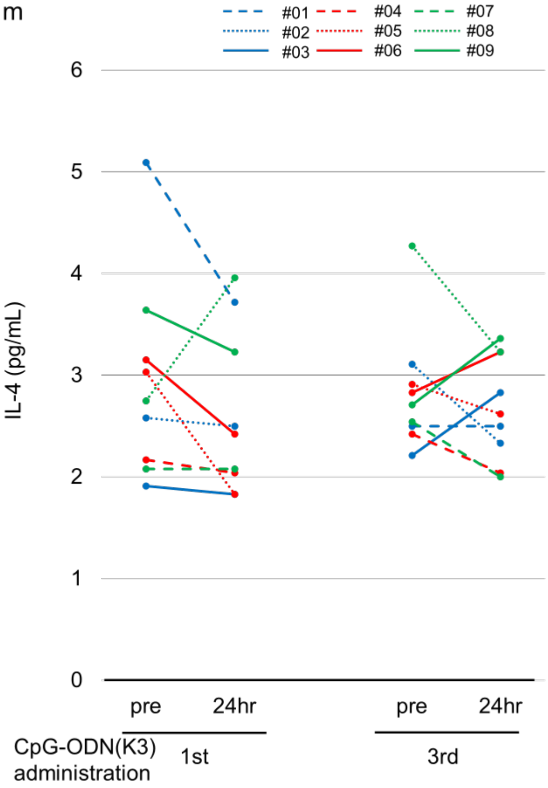
#01
#02
#03
#04
#05
#06
#07
#08
#09
6
5
4
3
2
1
0
IL-4 (pg/mL)
pre
24hr
pre
24hr
CpG-ODN(K3)
administration
1st
3rd

n

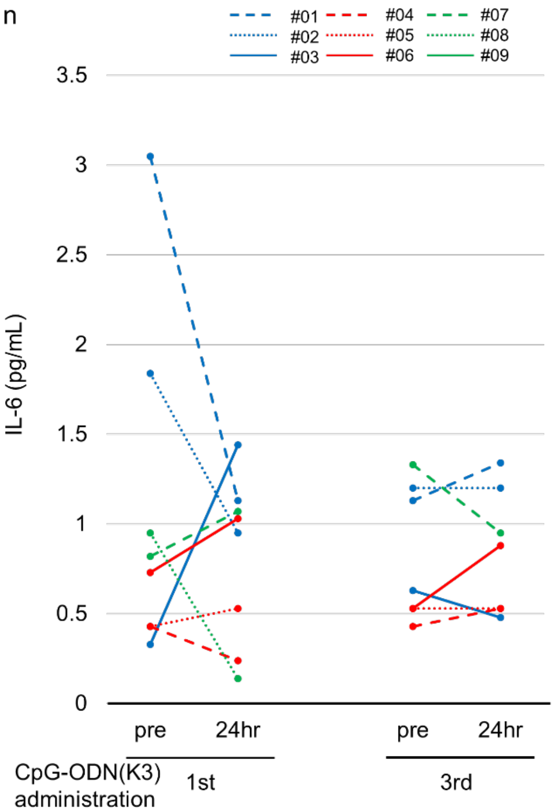
#01
#02
#03
#04
#05
#06
#07
#08
#09
3.5
3
2.5
2
1.5
1
0.5
0
IL-6 (pg/mL)
pre
24hr
pre
24hr
CpG-ODN(K3) administration
1st
3rd

o

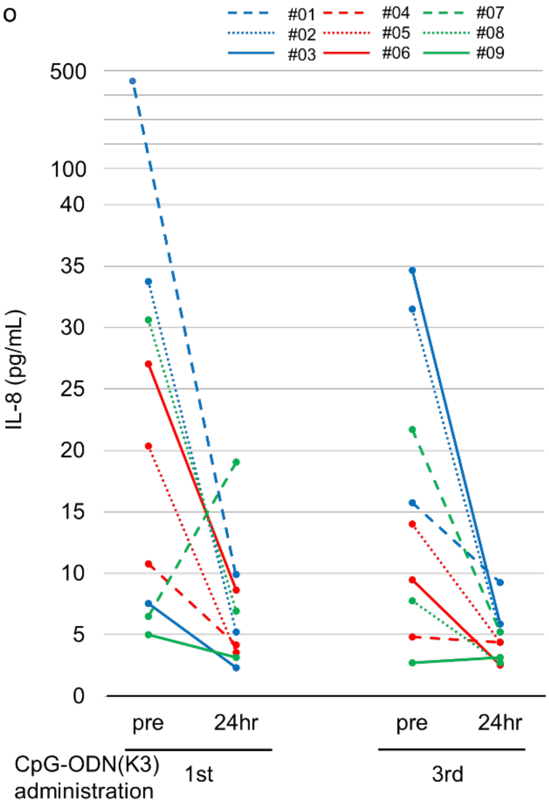
#01
#02
#03
#04
#05
#06
#07
#08
#09
500
100
40
35
30
25
20
15
10
5
0
IL-8 (pg/mL)
pre
24hr
pre
24hr
CpG-ODN(K3) administration
1st
3rd

p

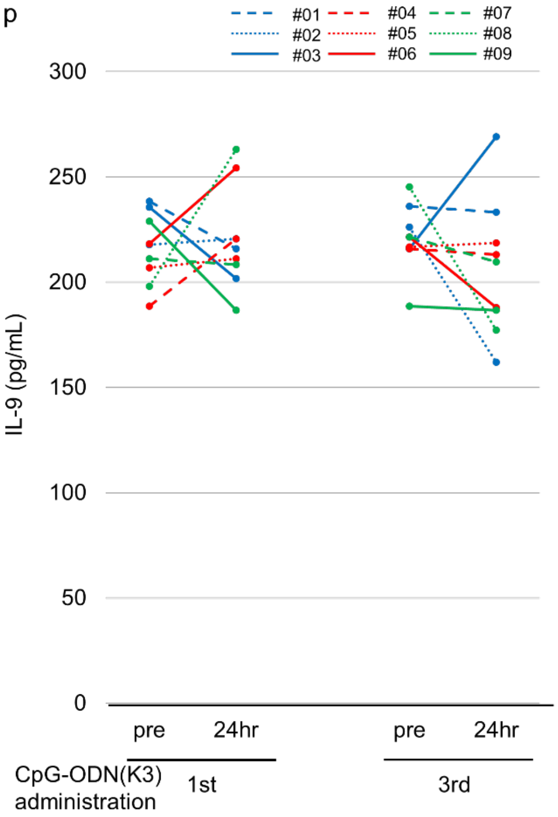
#01
#02
#03
#04
#05
#06
#07
#08
#09
300
250
200
150
100
50
0
IL-9 (pg/mL)
pre
24hr
pre
24hr
CpG-ODN(K3) administration
1st
3rd

q

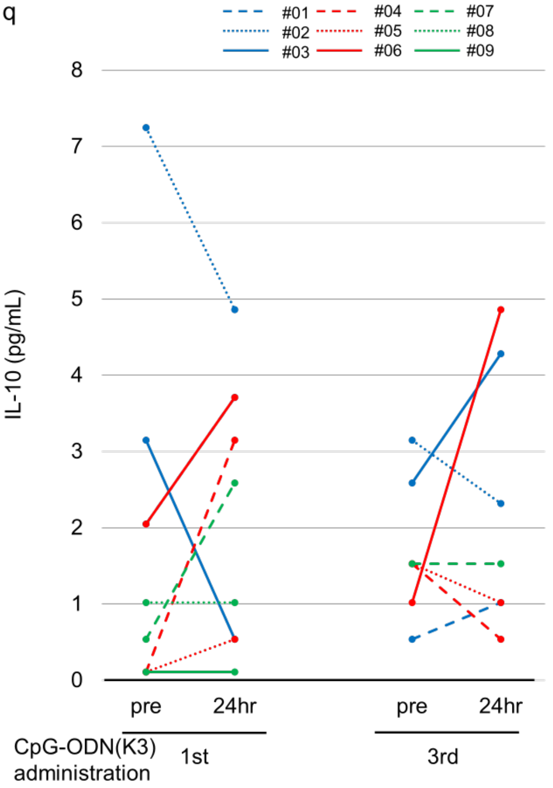
#01
#02
#03
#04
#05
#06
#07
#08
#09
8
7
6
5
4
3
2
1
0
IL-10 (pg/mL)
pre
24hr
pre
24hr
CpG-ODN(K3) administration
1st
3rd

r

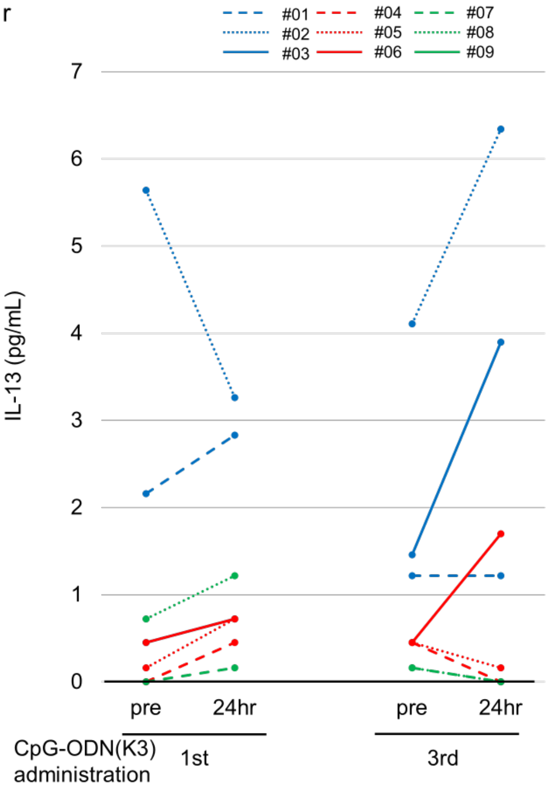

#01
#02
#03
#04
#05
#06
#07
#08
#09
7
6
5
4
3
2
1
0
IL-13 (pg/mL)
pre
24hr
pre
24hr
CpG-ODN(K3)
administration
1st
3rd

s

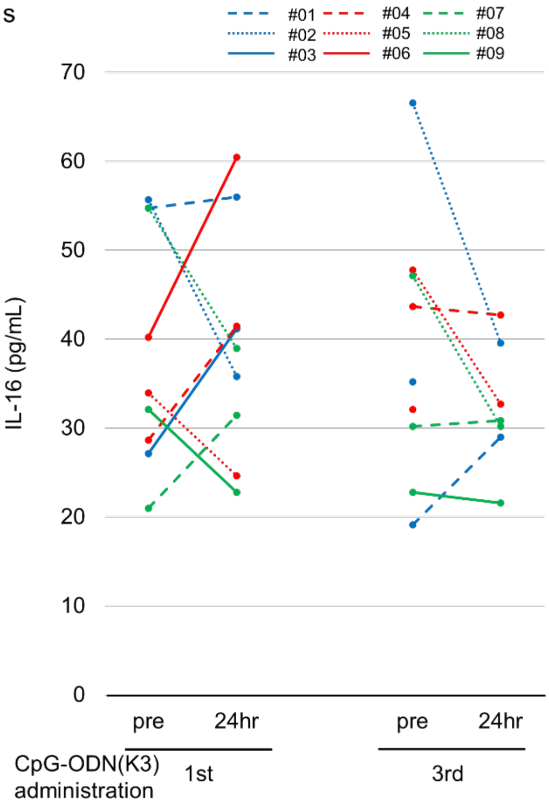

#01
#02
#03
#04
#05
#06
#07
#08
#09
70
60
50
40
30
20
10
0
IL-16 (pg/mL)
pre
24hr
pre
24hr
CpG-ODN(K3)
administration
1st
3rd

t

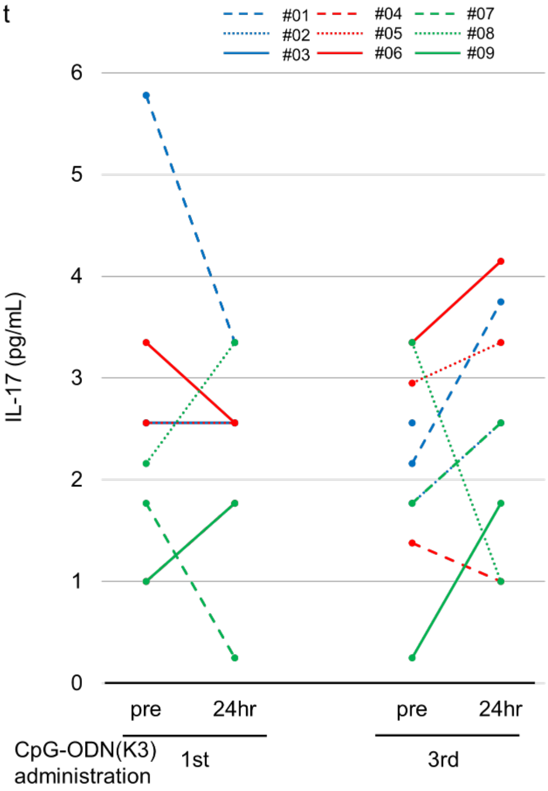
#01
#02
#03
#04
#05
#06
#07
#08
#09
6
5
4
3
2
1
0
IL-17 (pg/mL)
pre
24hr
pre
24hr
CpG-ODN(K3) administration
1st
3rd

u

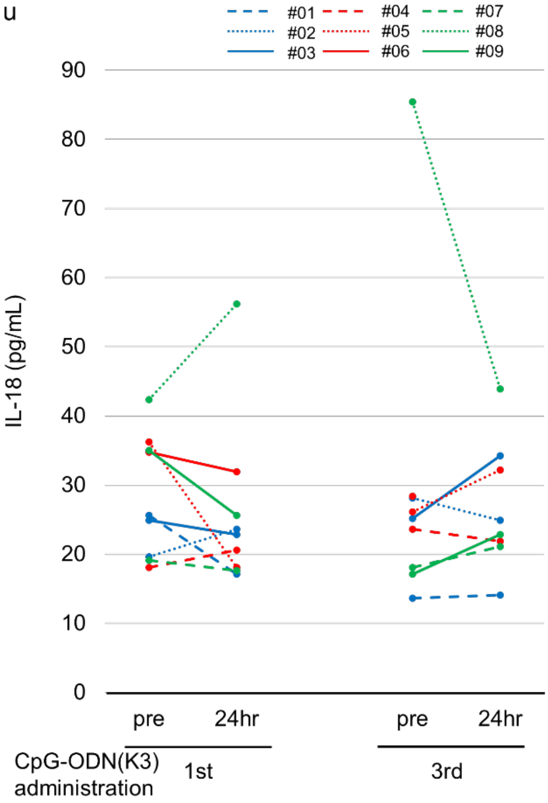
#01
#02
#03
#04
#05
#06
#07
#08
#09
90
80
70
60
50
40
30
20
10
0
IL-18 (pg/mL)
pre
24hr
pre
24hr
CpG-ODN(K3) administration
1st
3rd

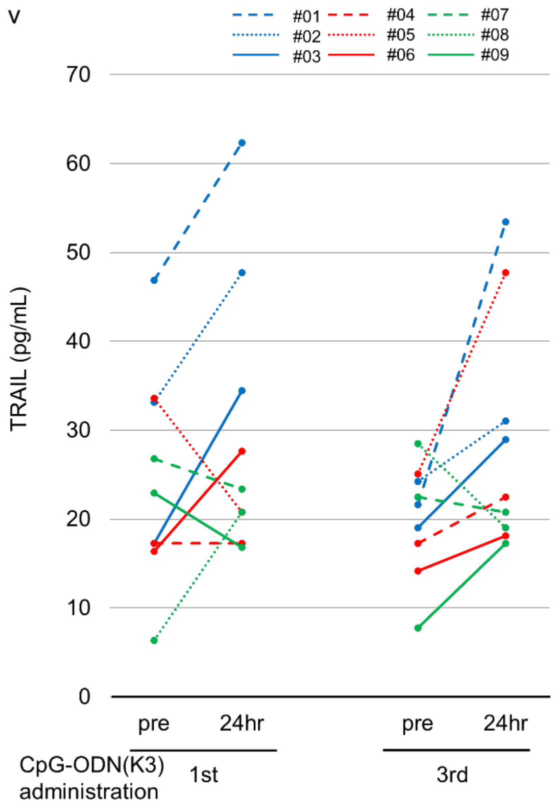
v
#01
#02
#03
#04
#05
#06
#07
#08
#09
TRAIL (pg/mL)
70
60
50
40
30
20
10
0
pre
24hr
pre
24hr
CpG-ODN(K3) administration
1st
3rd

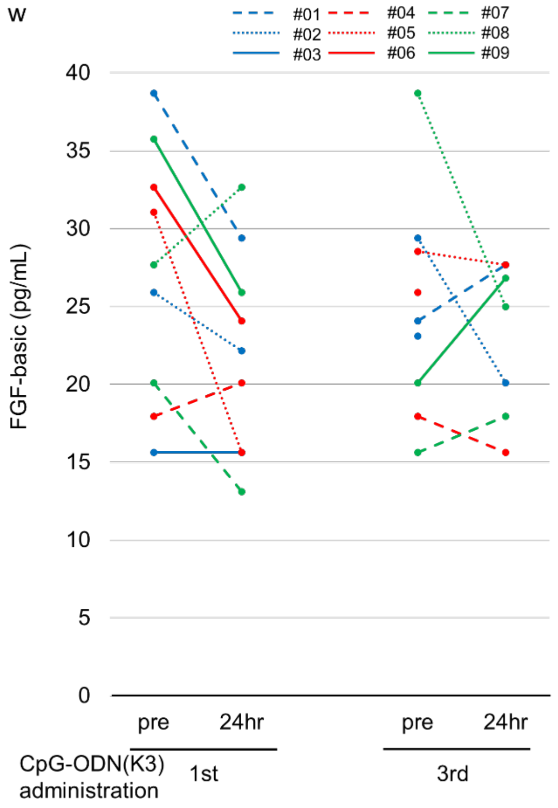
w
#01
#02
#03
#04
#05
#06
#07
#08
#09
FGF-basic (pg/mL)
40
35
30
25
20
15
10
5
0
pre
24hr
pre
24hr
CpG-ODN(K3) administration
1st
3rd

x

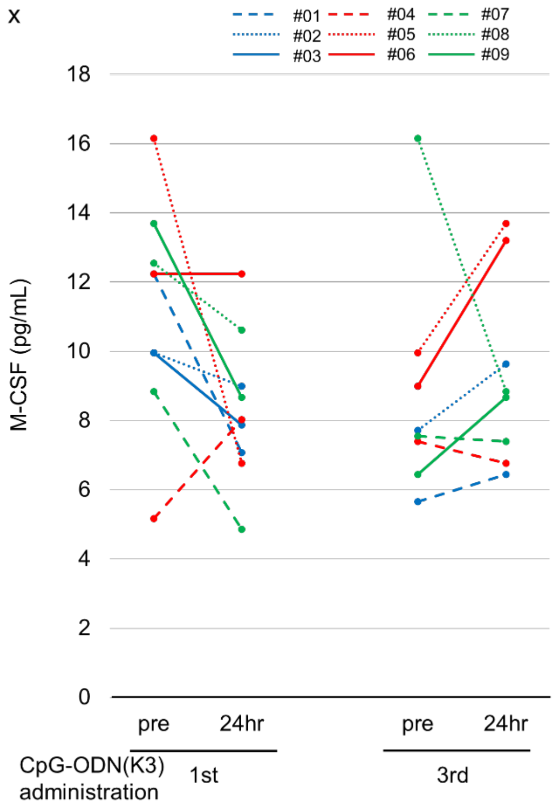
#01
#02
#03
#04
#05
#06
#07
#08
#09
18
16
14
12
10
8
6
4
2
0
M-CSF (pg/mL)
pre
24hr
pre
24hr
CpG-ODN(K3)
administration
1st
3rd

y

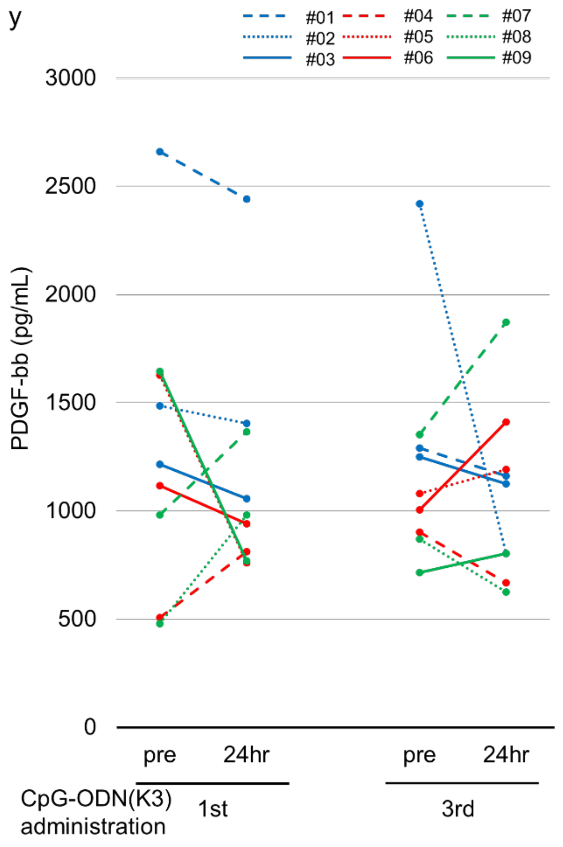
#01
#02
#03
#04
#05
#06
#07
#08
#09
3000
2500
2000
1500
1000
500
0
PDGF-bb (pg/mL)
pre
24hr
pre
24hr
CpG-ODN(K3)
administration
1st
3rd

z

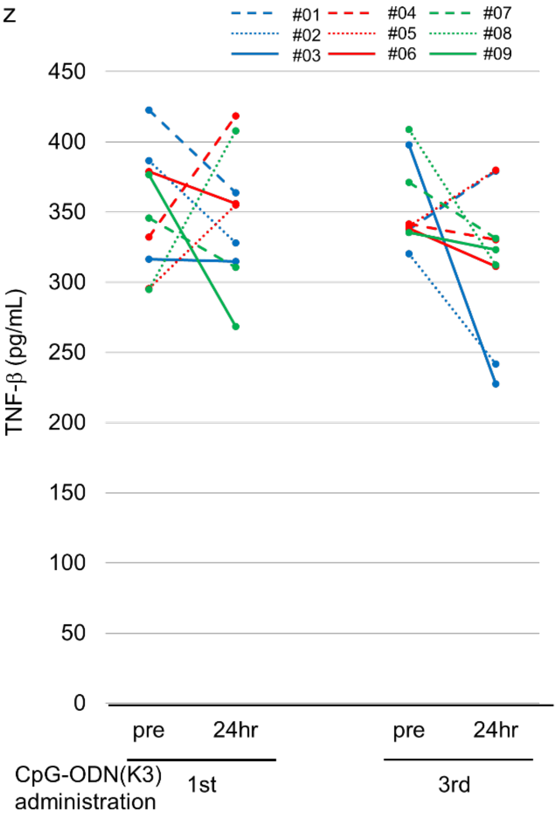
#01
#02
#03
#04
#05
#06
#07
#08
#09
450
400
350
300
250
200
150
100
50
0
TNF-β (pg/mL)
pre
24hr
pre
24hr
CpG-ODN(K3)
administration
1st
3rd

aa

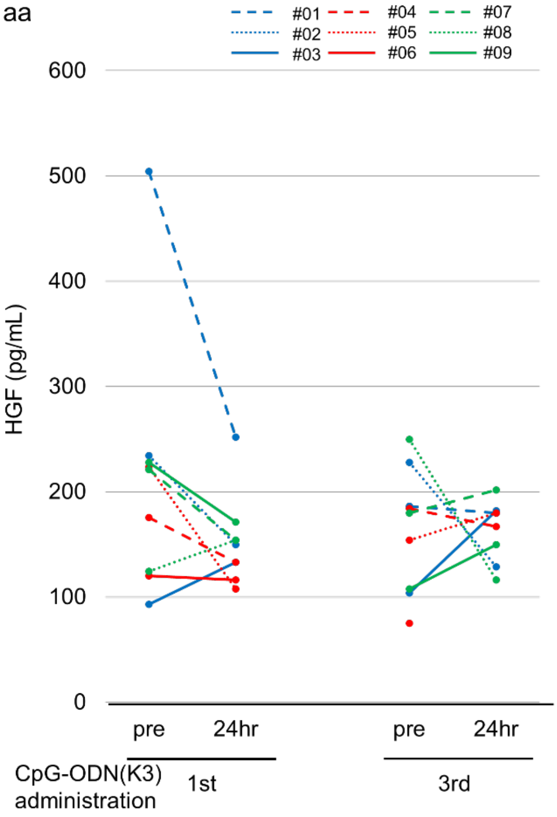
#01
#02
#03
#04
#05
#06
#07
#08
#09
600
500
400
300
200
100
0
HGF (pg/mL)
pre
24hr
pre
24hr
CpG-ODN(K3)
administration
1st
3rd

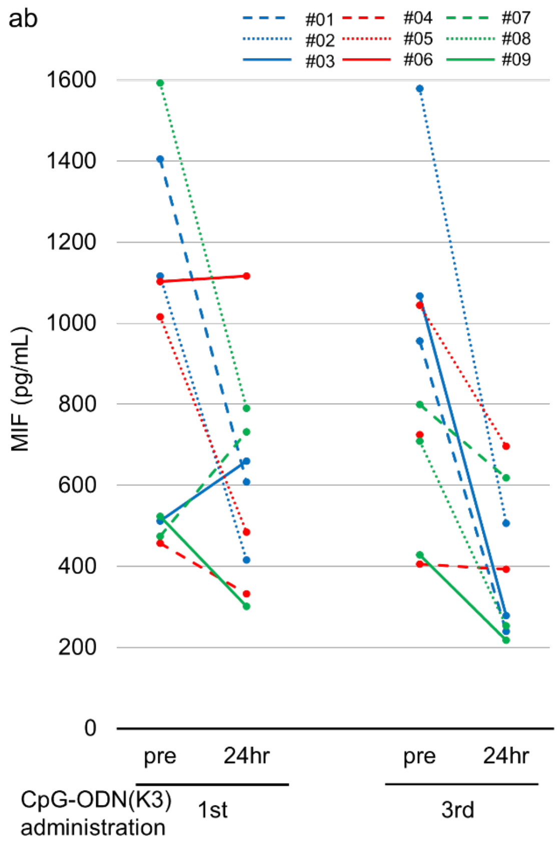

ab
#01
#02
#03
#04
#05
#06
#07
#08
#09
1600
1400
1200
1000
800
600
400
200
0
MIF (pg/mL)
pre
24hr
pre
24hr
CpG-ODN(K3)
administration
1st
3rd

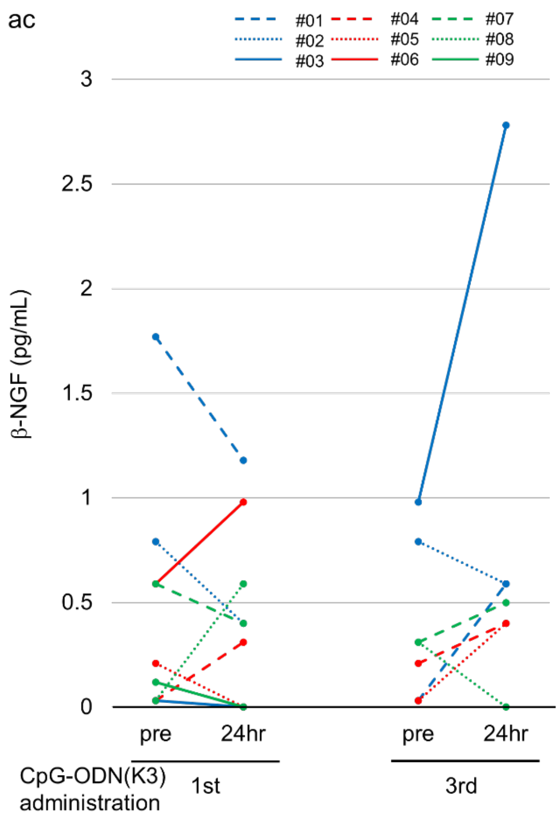

ac
#01
#02
#03
#04
#05
#06
#07
#08
#09
3
2.5
2
1.5
1
0.5
0
β-NGF (pg/mL)
pre
24hr
pre
24hr
CpG-ODN(K3)
administration
1st
3rd

ad

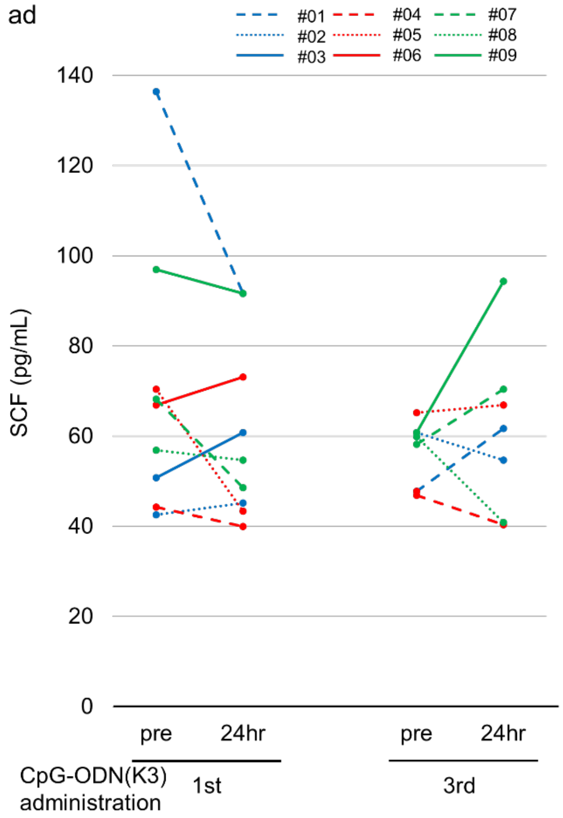
#01
#02
#03
#04
#05
#06
#07
#08
#09
140
120
100
80
60
40
20
0
SCF (pg/mL)
pre
24hr
pre
24hr
CpG-ODN(K3)
administration
1st
3rd

ae

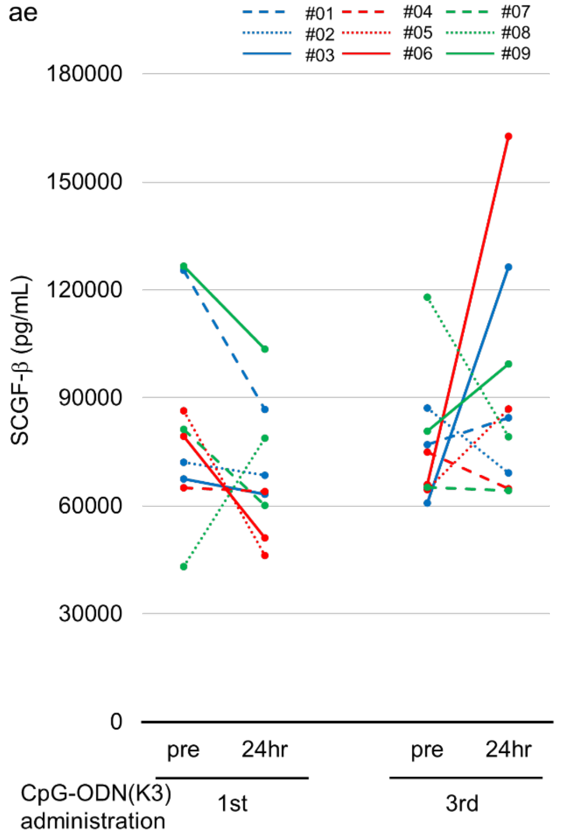
#01
#02
#03
#04
#05
#06
#07
#08
#09
180000
150000
120000
90000
60000
30000
0
SCGF-β (pg/mL)
pre
24hr
pre
24hr
CpG-ODN(K3)
administration
1st
3rd

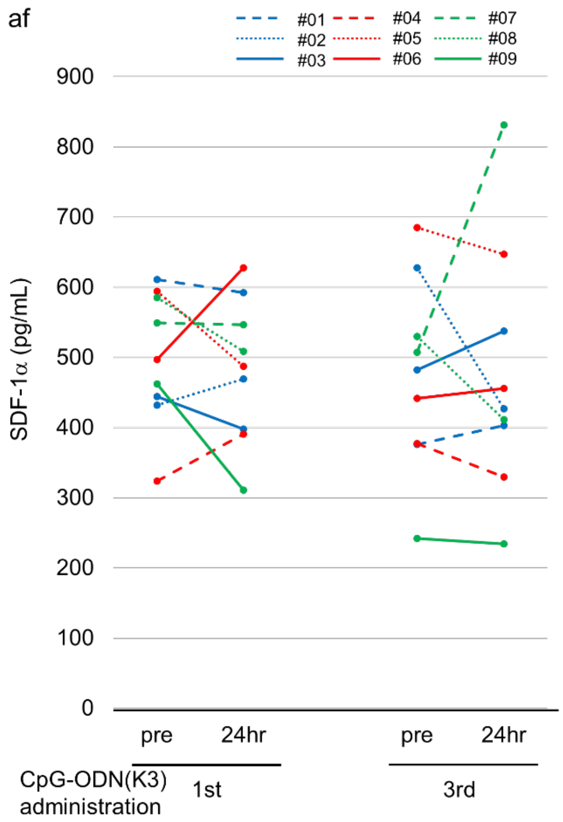
af
#01
#02
#03
#04
#05
#06
#07
#08
#09
900
800
700
600
500
400
300
200
100
0
SDF-1α (pg/mL)
pre
24hr
pre
24hr
CpG-ODN(K3)
administration
1st
3rd

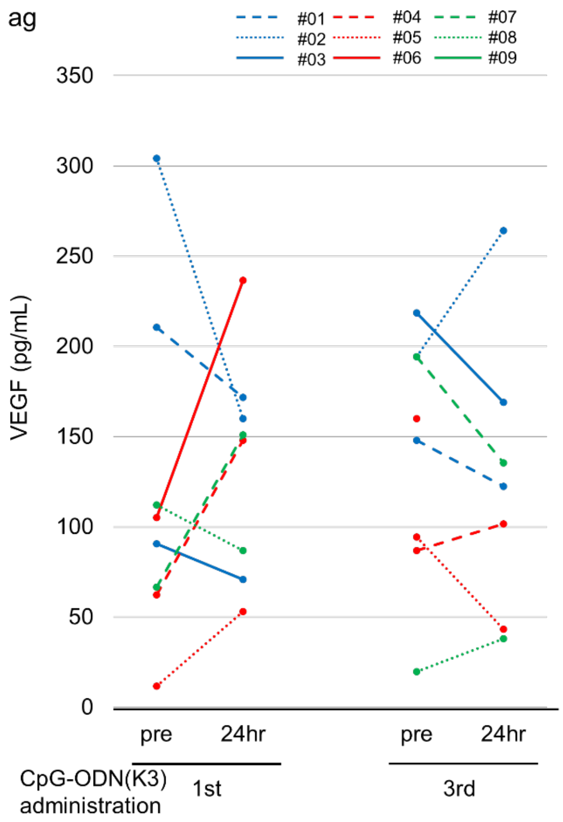
ag
#01
#02
#03
#04
#05
#06
#07
#08
#09
350
300
250
200
150
100
50
0
VEGF (pg/mL)
pre
24hr
pre
24hr
CpG-ODN(K3)
administration
1st
3rd

Supplementary Figure S3. Gating strategy for immune phenotyping and functional characteristics of T cells.

a. $CD4^+$ and $CD8^+$ T cell subpopulations were determined based on CCR7 and CD45RA expression: naive (CCR7+CD45RA+), central memory (CM) (CCR7+CD45RA-), effector memory (EM) (CCR7-CD45RA-), and terminally differentiated effector memory (TEMRA) (CCR7-CD45RA+) cells. Analysis of functional characteristics in $CD4^+$ T cells. Th1 T cells and regulatory T cells in $CD4^+$ T cells were gated based on T-bet or Foxp3 expression. Analysis of T-bet expression in $CD8^+$ T cells. **b.** Results of the analyses of immune phenotypes and functional characteristics with PBMCs collected from two healthy subjects

a

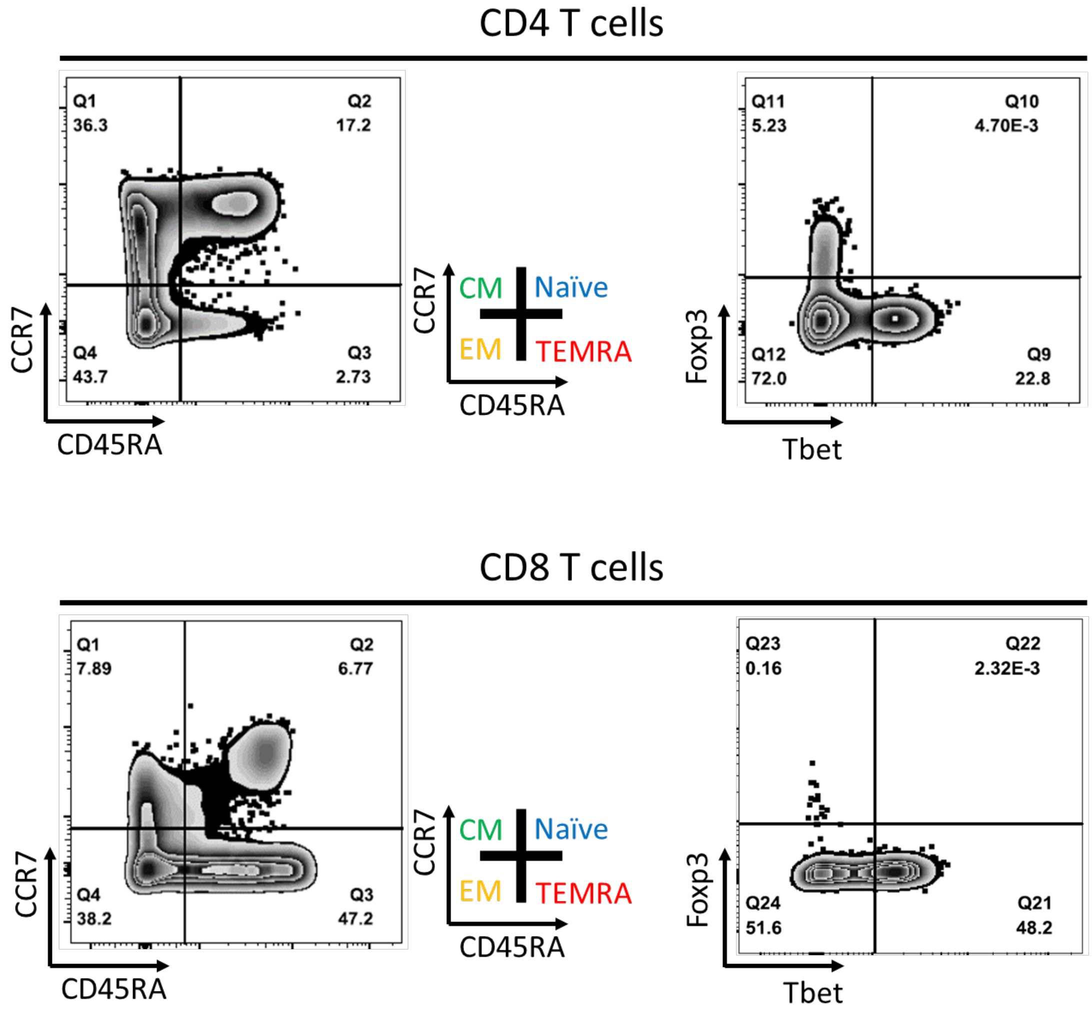

b

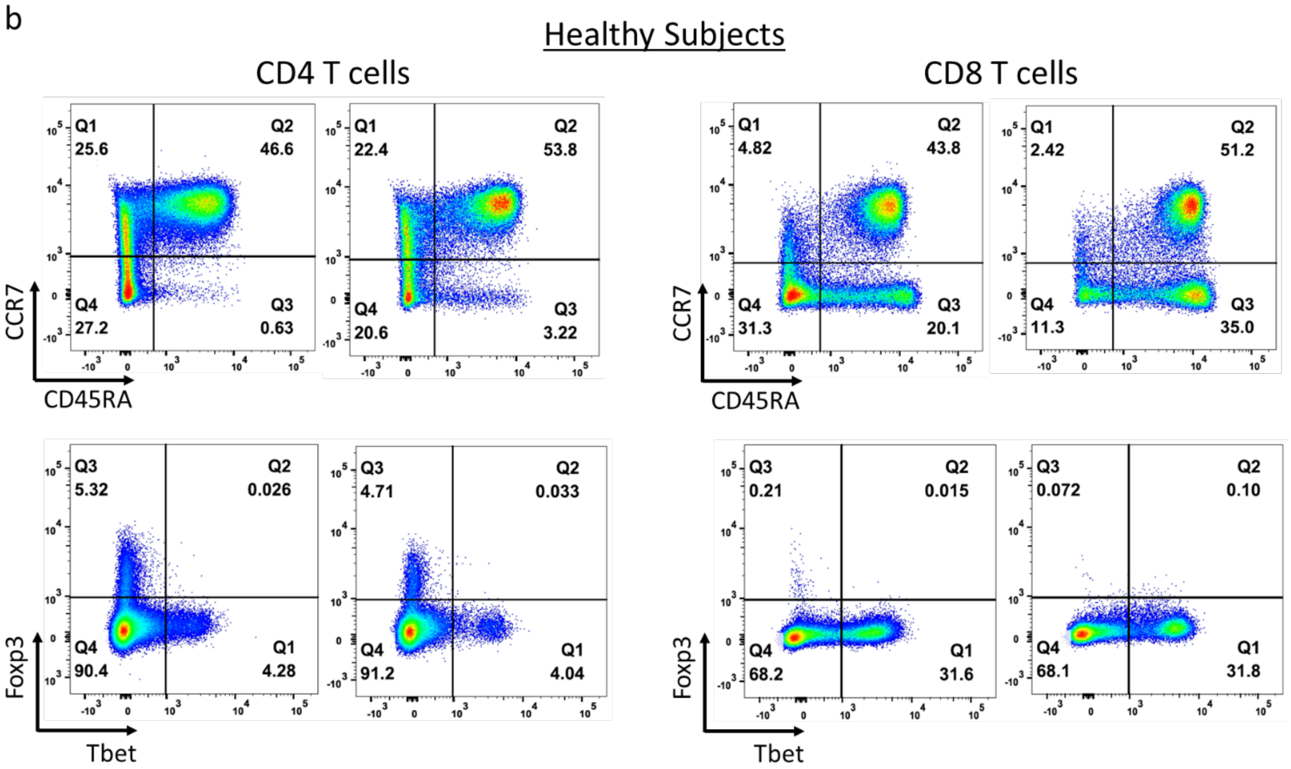

Healthy Subjects
CD4 T cells
CD8 T cells
Q1
25.6
Q2
46.6
Q4
27.2
Q3
0.63
Q1
22.4
Q2
53.8
Q4
20.6
Q3
3.22
CCR7
CD45RA
Q1
4.82
Q2
43.8
Q4
31.3
Q3
20.1
Q1
2.42
Q2
51.2
Q4
11.3
Q3
35.0
Q3
5.32
Q2
0.026
Q4
90.4
Q1
4.28
Q3
4.71
Q2
0.033
Q4
91.2
Q1
4.04
Foxp3
Tbet
Q3
0.21
Q2
0.015
Q4
68.2
Q1
31.6
Q3
0.072
Q2
0.10
Q4
68.1
Q1
31.8

Supplementary Figure S4. Foxp3 expression in effector memory of CD4^{+} T cells in individual cases.

At baseline, patients with T-bet predominance (**left**), T-bet / Foxp3 equivalent (**middle**), and Foxp3 predominance (**right**) in CD4^{+} T cells were classified into groups 1, 2, and 3, respectively. Blue lines, dose level 0; red lines, dose level 1; and green lines, dose level 2

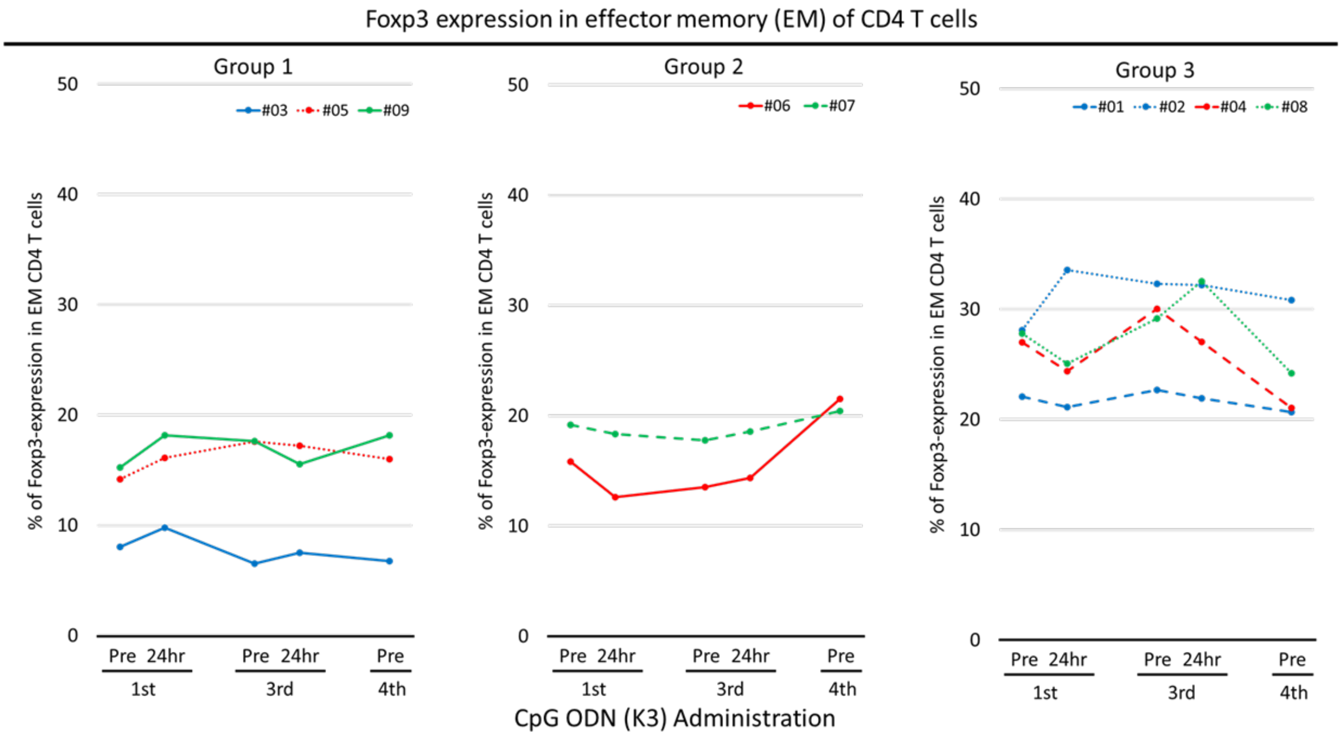

Supplement: Supplementary file 2 — Additional file 2: Supplementary Fig. S1. Kaplan–Meier curves for progression-free survival (PFS). Supplementary Fig. S2. Analysis of chemokines and cytokines. Supplementary Fig. S3. Gating strategy for immune phenotyping and functional characteristics of T cells. Supplementary Fig. S4. Foxp3 expression in effector memory of CD4+ T cells in individual cases. [file 12885_2022_9818_MOESM2_ESM.pdf]
